# Chemical Synthesis and Biological Evaluation of 3-Substituted Estrone/Estradiol Derivatives as 17β-Hydroxysteroid Dehydrogenase Type 1 Inhibitors Acting via a Reverse Orientation of the Natural Substrate Estrone

**DOI:** 10.3390/molecules28020632

**Published:** 2023-01-07

**Authors:** Adrien Djiemeny Ngueta, Jenny Roy, René Maltais, Donald Poirier

**Affiliations:** 1 Laboratory of Medicinal Chemistry, Endocrinology and Nephrology Unit, CHU de Québec Research Center—Université Laval, Quebec, QC G1V 4G2, Canada; 2 Department of Molecular Medicine, Faculty of Medicine, Université Laval, Quebec, QC G1V 0A6, Canada

**Keywords:** steroid, chemical synthesis, inhibitor, 17β-HSD1, estrogen

## Abstract

Estradiol (E2) plays an important role in the progression of diseases such as breast cancer and endometriosis. Inhibition of 17β-hydroxysteroid dehydrogenase type 1 (17β-HSD1), the enzyme that catalyzes the last step in the biosynthesis of the estrogenic hormone E2, therefore constitutes an interesting approach for the treatment of these two estrogen-dependent diseases. In order to obtain new inhibitors of 17β-HSD1, the impact of a *m*-carbamoylphenyloxy group at position three of an estrane nucleus was evaluated by preparing three derivatives of estrone (E1) and E2 using a microwave-assisted synthesis of diaryl ethers. Their inhibitory activity was addressed on two cell lines (T-47D and Z-12) representative of breast cancer and endometriosis, respectively, but unlike T-47D cells, Z-12 cells were not found suitable for testing potential 17β-HSD1 inhibitors. Thus, the addition of the *m*-carbamoylphenyl group at C3 of E1 (compound **5**) did not increase the inhibition of E1 to E2 transformation by 17β-HSD1 present in T-47D cells (IC_50_ = 0.31 and 0.21 μM for **5** and E1, respectively), and this negative effect was more obvious for E2 derivatives **6** and **10** (IC_50_ = 1.2 and 1.3 μM, respectively). Molecular docking allowed us to identify key interactions with 17β-HSD1 and to highlight these new inhibitors’ actions through an opposite orientation than natural enzyme substrate E1′s classical one. Furthermore, molecular modeling experiments explain the better inhibitory activity of E1-ether derivative **5,** as opposed to the E2-ether derivatives **6** and **10**. Finally, when tested on T-47D and Z-12 cells, compounds **5**, **6** and **10** did not stimulate the proliferation of these two estrogen-dependent cell lines. In fact, they reduced it.

## 1. Introduction

**Figure 1:**
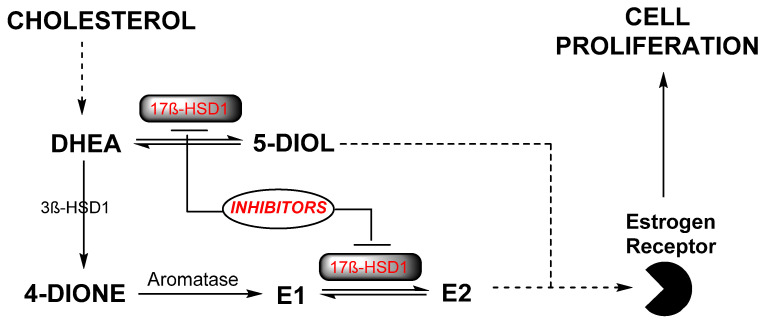
Pathways involved in the formation of potent estrogen estradiol (E2) as well as weak estrogens estrone (E1) and 5-androstene-3β,17β-diol (5-DIOL) from dehydroepiandrosterone (DHEA). HSD: Hydroxysteroid dehydrogenase; 4-DIONE: 4-androstene-3,17-dione.

Estrogenic steroid hormones play an important role in the development of the genitals and sex characteristics, as well as for the maintenance of bone mass in women [1]. However, these hormones are also involved in so-called estrogen-dependent diseases (EDDs), such as breast cancer and endometriosis, through the estrogen receptor (ER) expressed in breast tumors and endometrium tissues [2,3,4]. Breast cancer is the leading cancer in women worldwide, with 2.3 million cases diagnosed in 2020, according to the World Health Organization [5]. In addition, endometriosis is a disease characterized by the development of tissue similar to the uterine lining, outside the uterus, thus causing pain and/or infertility [6], and this disease affects nearly 10% of women and girls of childbearing age, or 190 million people, worldwide [7]. It is now well recognized that estrogenic hormones are important stimulators of EDDs. In women, ovaries are largely responsible for the production of estrogens, but the adrenal gland also secretes large quantities of steroid precursors, which are converted into active estrogens in the peripheral tissues (Figure 1) [8,9]. The control of estrogen production would therefore be an interesting approach for the treatment of breast cancer and endometriosis.

**Figure 2:**
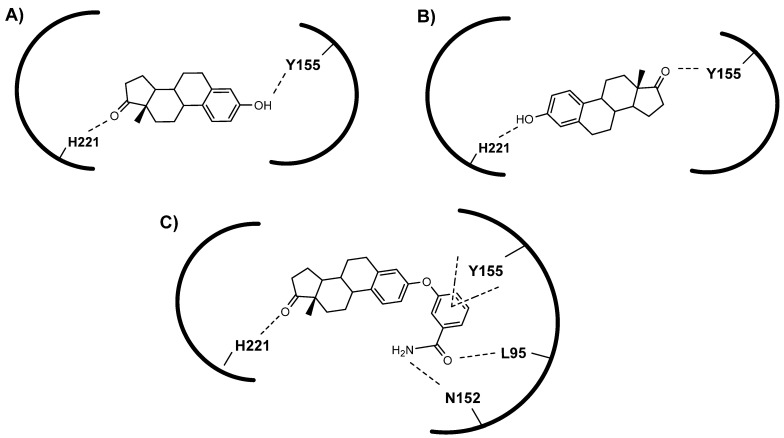
Illustrations of key interactions predicted in binary complexes of 17β-HSD1 (catalytic binding site) and natural substrate E1 positioned in a reversed side (**A**), E1 positioned in a normal side (**B**) and predicted inhibitor **5** (**C**).

17β-Hydroxysteroid dehydrogenase type 1 (17β-HSD1) catalyzes the last step in estrogen formation and is involved in the development of EDDs [10,11,12,13]. Thus, by decreasing the concentration of active estrogens, an inhibitor of 17β-HSD1 activity could complement the use of an anti-estrogen (ER antagonist) in the treatment of breast cancer, as well as for the treatment of endometriosis. Different strategies have therefore been used so far to develop 17β-HSD1 inhibitors [14,15,16,17,18]. More recently, and by resolving the binary and ternary crystal structures of 17β-HSD1 in complex with estrone (E1) as well as with the cofactor NADP^+^ and E1 [19], some critical enzyme-substrate-cofactor interactions have been observed. Notably, the structural analysis of these two complexes showed interesting interactions between the His-221 imidazole residue and the natural substrate E1 positioned in a reversed side, leading to the formation of a dead-end complex (Figure 2A) [19], as opposed to the classic complex between 17β-HSD1 and E1 positioned in normal side (Figure 2B), which allows the formation of E2 [20]. From modeling work, the authors proposed that compound **5** could inhibit 17β-HSD1 through the formation of a dead-end-type complex (Figure 2C) [19].

**Figure 3:**
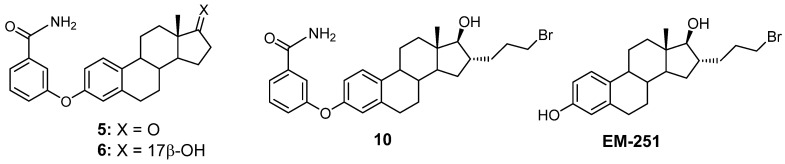
New E1/E2 derivatives modified at position 3 (compounds **5**, **6** and **10**) synthesized as potential inhibitors of 17β-HSD1 and the known inhibitor EM-251 (compound **23** in [26]).

In continuation of our work on 17β-HSD1 inhibitors [21,22,23,24,25], we have synthesized and characterized the E1 derivative, compound **5**, as well as two E2 derivatives, compounds **6** and **10** (Figure 3). Compound **10** is a hybrid molecule of ether-derivative **6** and EM-251, the latter being a known inhibitor of 17β-HSD1 [26,27,28]. The ability of compounds **5**, **6** and **10** to inhibit 17β-HSD1, as well as their impact on cell proliferation, were evaluated using breast cancer T-47D cells and endometriosis Z-12 cells. Thus, the aim of this study is to experimentally validate the reverse orientation hypothesis of an enzyme ligand in order to design a novel kind of 17β-HSD1 inhibitor to treat EDDs.

## 2. Materials and Methods

### 2.1. Chemistry (General)

Chemical reagents were purchased from Sigma-Aldrich Canada Ltd. (Oakville, ON, Canada). The usual solvents were obtained from Fisher Scientific (Montreal, QC, Canada) and used as received, while anhydrous dimethylsulfoxide (DMSO) was obtained from Sigma-Aldrich. Starting materials, estrone (**1**) and estradiol (**2**) were purchased from Sigma-Aldrich, while compound **7** and EM-251 were synthesized, as previously described [26]. Thin-layer chromatography (TLC) and flash-column chromatography were performed on 0.20-mm silica gel 60 F254 plates (E. Merck KG; Darmstadt, Germany) and with 230–400 mesh ASTM silica gel 60 (Silicycle, Quebec, QC, Canada), respectively. Microwave experiments were conducted in a Biotage Initiator microwave instrument (Charlotte, NC, USA). Infrared (IR) spectra were recorded on a Horizon MB 3000 ABB FTIR spectrometer (Quebec, QC, Canada) and only the important bands were reported in cm^−1^. Nuclear magnetic resonance (NMR) spectra were recorded at 400 MHz for ^1^H and 100.6 MHz for ^13^C on a Bruker Avance 400 digital spectrometer (Billerica, MA, USA). Chemical shifts (δ) were expressed in ppm and referenced to chloroform (7.26 and 77.0 ppm for ^1^H and ^13^C NMR, respectively). Low-resolution mass spectra (LRMS) were expressed in *m/z* and recorded on a Shimadzu Prominence apparatus (Kyoto, Japan) equipped with a LCMS-2020 mass spectrometer (APCI probe). High-resolution mass spectra (HRMS) of final compounds were provided by Pierre Audet at the Université Laval Chemistry Department (Quebec, QC, Canada). The purity of the final compounds to be tested was determined with a Shimadzu HPLC apparatus equipped with an SPD-M20A photodiode array detector, a Setima HPLC18 reversed-phase column (250 mm × 4.6 mm) and using a solvent gradient of MeOH:water from 70:30 to 100:0 on 30 min run. The wavelength of the UV detector was 210 nm.

### 2.2. Chemistry (Chemical Synthesis)

#### 2.2.1. Synthesis of 3-(3-Cyanophenyloxy)-estra-1,3,5(10)-trien-17-one (**3**)

Estrone (**1**) (150 mg, 0.555 mmol), DMSO (2.5 mL), 3-fluorobenzonitrile (135 mg, 1.11 mmol) and potassium carbonate (153 mg, 1.11 mmol) were added in a microwave tube. Using a microwave power of 300 W, we ramped the temperature from room temperature to the boiling point of DMSO, which took ~5 min, and then held DMSO at a boil for 1 min. After the reaction was cooled to room temperature, the mixture was poured into water and extracted with EtOAc. The organic phase was dried over MgSO_4_, filtered, and evaporated under reduced pressure. The crude product was purified by flash chromatography using as eluent a mixture of CH_2_Cl_2_/MeOH (95:5) to afford 60 mg (30%) of compound **3**. IR υ: 2230 (C≡N), 1736 (C=O) and 1582 (C=C). ^1^H NMR (400 MHz, CDCl_3_) δ: 0.94 (s, 18-CH_3_), 1.40–2.60 (m, unassigned CH and CH_2_), 2.90 (m, 6-CH_2_), 6.77 (s, 1H aryl), 6.82 (d, *J* = 8.3 Hz, 1H aryl), 7.16 (s, 1H aryl), 7.21–7.43 (m, 4H aryl). LRMS for C_25_H_26_NO_2_ [M+H]^+^: Calculated 372.20 and found 372.15 *m/z*.

#### 2.2.2. Synthesis of 3-(3-Cyanophenyloxy)-estra-1,3,5(10)-trien-17β-ol (**4**)

As reported in Section 2.2.1, a solution of estradiol (**2**) (100 mg, 0.367 mmol) in DMSO (2.5 mL), 3-fluorobenzonitrile (90 mg, 0.734 mmol) and potassium carbonate (101 mg, 0.734 mmol) was reacted in a microwave tube and the crude product was purified by flash chromatography, using as eluent a mixture of hexanes/EtOAc (8:2) to afford 97 mg (71%) of compound **4**. IR υ: 3564 (OH), 2230 (C≡N) and 1574 (C=C).^1^H NMR (400 MHz, CDCl_3_) δ: 0.81 (s, 18-CH_3_), 1.15–2.36 (m, unassigned CH and CH_2_), 2.85 (m, 6-CH_2_), 3.75 (t, *J* = 8.3 Hz, 17α-CH), 6.74 (d, *J* = 2.2 Hz, 1H aryl), 6.80 (dd, *J* = 2.4 Hz and *J* = 8.4 Hz, 1H aryl), 7.17 (s, 1H aryl), 7.21–7.42 (m, 4H aryl). ^13^C NMR (101 MHz, CDCl_3_) δ: 11.1, 23.1, 26.2, 27.0, 29.6, 30.6, 36.7, 38.6, 43.2, 44.1, 50.0, 81.8, 113.3, 117.1, 118.4, 120.0, 120.6, 122.5, 126.0, 127.1, 130.5, 137.1, 139.1, 153.0, 158.6. LRMS for C_25_H_26_NO [M+H-H_2_O]^+^: Calculated 356.20 and found 356.10 *m/z*.

#### 2.2.3. Synthesis of 3-(3-Carbamoylphenyloxy)-estra-1,3,5(10)-trien-17-one (**5**)

Compound **3** (53 mg, 0.146 mmol) was dissolved in a mixture of water:THF/1:3 (8 mL). Acetamide (260 mg, 4.39 mmol) and PdCl_2_ (26 mg, 0.146 mmol) were added, and the mixture was stirred at room temperature overnight. THF was evaporated and the mixture was poured into water and extracted with EtOAc. The organic phase was dried over MgSO_4,_ filtered, and evaporated under reduced pressure. The crude product was purified by flash chromatography, using as an eluent a mixture of CH_2_Cl_2_/MeOH (95:5), to afford 52 mg (91%) of compound **5**. IR υ: 3400 and 3340 (NH_2_), 3200 (OH), 1728 (C=O), 1659 (NC=O) and 1574 (C=C). ^1^H NMR (400 MHz, CDCl_3_) δ: 0.94 (s, 18-CH_3_), 1.40–2.55 (m, unassigned CH and CH_2_), 2.88 (m, 6-CH_2_), 5.59 (m, 1H of NH_2_), 6.03 (m, 1H of NH_2_), 6.76 (s, 1H aryl), 6.81 (d, *J* = 8.3 Hz, 1H aryl), 7.17 (d, *J* = 7.8 Hz, 1H aryl), 7.27 (s, 1H aryl), 7.37–7.50 (m, 2H aryl). ^13^C NMR (101 MHz, CDCl_3_) δ: 13.8, 21.6, 25.8, 26.4, 29.5, 31.5, 35.8, 38.1, 44.1, 48.0, 50.4, 116.8, 117.2, 119.4, 121.4, 121.9, 126.8, 129.9, 135.1, 135.5, 138.5, 154.2, 158.1, 168.9, 220.9. HRMS for C_25_H_28_NO_3_ [M+H]^+^: Calculated 390.2064 and found 390.2077 *m/z.* HPLC purity: 96.3% (RT = 15.2 min). (Supplementary Materials).

#### 2.2.4. Synthesis of 3-(3-Carbamoylphenyloxy)-estra-1,3,5(10)-trien-17β-ol (**6**)

As reported in Section 2.2.3, compound **4** (50 mg, 0.134 mmol), dissolved in a mixture of water:THF/1:3 (8 mL), was reacted with acetamide (237 mg, 4.02 mmol) and PdCl_2_ (23 mg, 0.134 mmol). The crude product was purified by flash chromatography, using as eluent a mixture of CH_2_Cl_2_/MeOH (97:3), to afford 47 mg (89%) of compound **6**. IR υ: 3370 and 3333 (NH_2_), 3202 (OH), 1659 (NC=O) and 1574 (C=C). ^1^H NMR (400 MHz, CDCl_3_) δ: 0.80 (s, 18-CH_3_), 1.10–2.38 (m, unassigned CH and CH_2_), 2.83 (m, 6-CH_2_), 3.74 (t, *J* = 8.3 Hz, 17α-CH), 5.91 (m, 1H of NH_2_), 6.09 (m, 1H of NH_2_), 6.73 (s, 1H aryl), 6.80 (d, *J* = 7.9 Hz, 1H aryl), 7.16 (s, 1H aryl), 7.25–7.50 (m, 4H aryl). ^13^C NMR (101 MHz, CDCl_3_) δ: 11.0, 23.1, 26.2, 27.1, 29.6, 30.6, 36.7, 38.6, 43.2, 44.1, 50.0, 81.8, 116.6, 117.1, 119.4, 121.4, 121.9, 126.8, 129.9, 135.0, 136.1, 138.7, 154.0, 158.2, 168.9. HRMS for C_25_H_30_NO_3_ [M+H]^+^: Calculated 392.2220 and found 392.2229 *m/z*. HPLC purity: 97.1% (RT = 16.1 min). (Supplementary Materials).

#### 2.2.5. Synthesis of 3-(3-Cyanophenyloxy)-16α-(3-hydroxypropyl)-estra-1,3,5(10)-trien-17β-ol (**8**)

As reported in Section 2.2.1, a solution of compound **7** [26] (110 mg, 0.332 mmol) in DMSO (10 mL), 3-fluorobenzonitrile (80 mg, 0.660 mmol) and potassium carbonate (91 mg, 0.660 mmol) was reacted in a microwave tube and the crude product was purified by flash chromatography, using as eluent a mixture of hexane/EtOAc (7:3) to afford 100 mg (70%) of compound **8**. ^1^H NMR (400 MHz, CDCl_3_) δ: 0.84 (s, 18-CH_3_), 1.15–2.35 (m, unassigned CH and CH_2_), 2.84 (m, 6-CH_2_), 3.32 (d, *J* = 7.5 Hz, 17α-CH), 3.69 (t, *J* = 5.9 Hz, CH_2_OH), 6.75 (s, 1H aryl), 6.80 (dd, *J_1_* = 2.3 Hz and *J_2_* = 8.4 Hz, 1H aryl), 7.17 (s, 1H aryl) 7.20–7.42 (m, 4H aryl). LRMS for C_28_H_32_NO_2_ [M+H-H_2_O]^+^: Calculated 414.24 and found 414.20 *m/z*.

#### 2.2.6. Synthesis of 3-(3-Carbamoylphenyloxy)-16α-(3-hydroxypropyl)-estra-1,3,5(10)-trien-17β-ol (**9**)

As reported in Section 2.2.3, compound **8** (100 mg, 0.231 mmol), dissolved in a mixture of water:THF/1:3 (12 mL), was reacted with acetamide (409 mg, 6.94 mmol) and PdCl_2_ (41 mg, 0.231 mmol). The crude product was purified by flash chromatography, using as eluent a mixture of hexane/acetone (1:1), to afford 70 mg (67%) of compound **9**. ^1^H NMR (400 MHz, CDCl_3_) δ: 0.82 (s, 18-CH_3_), 1.10–2.45 (m, unassigned CH and CH_2_), 2.81 (m, 6-CH_2_), 3.30 (s, 1H, 17α-CH), 3.69 (m, CH_2_OH), 4.10 (s, 1H of OH), 5.70 (m, 1H of NH_2_), 6.16 (m, 1H of NH_2_), 6.72 (s, 1H aryl), 6.78 (broad, 1H aryl), 7.15 (s, 1H aryl), 7.20–7.48 (m, 4H aryl).

#### 2.2.7. Synthesis of 3-(3-Carbamoylphenyloxy)-16α-(3-bromopropyl)-estra-1,3,5(10)-trien-17β-ol (**10**)

Compound **9** (50 mg, 0.111 mmol) was dissolved in 1,2-dibromoethane (5 mL), and triphenylphosphine (52 mg, 0.2 mmol) and tetrabutylammonium iodide (TBAI) (74 mg, 0.2 mmol) were added. The mixture was stirred at 60 °C overnight and the resulting solution was evaporated under reduced pressure. Purification by flash chromatography using hexane/acetone (75:25) provided 15 mg (26%) of compound **10**. IR υ: 3203 and 3672 (NH_2_), 3456 (OH), 1674 (NC=O) and 1574 (C=C). ^1^ H NMR (400 MHz, CDCl_3_) δ: 0.82 (s, 18-CH_3_), 1.20–2.40 (m, unassigned CH and CH_2_), 2.83 (m, 6-CH_2_), 3.30 (d, *J* = 5.7 Hz, 1H), 3.45 (broad, CH_2_Br), 5.72 (m, 1H of NH_2_), 6.05 (m, 1H of NH_2_), 6.73 (s, 1H aryl), 6.80 (d, *J* = 7.9 Hz, 1H aryl), 7.16 (d, *J* = 7.2 Hz, 1H aryl), 7.20–7.50 (m, 4H aryl). ^13^C NMR (101 MHz, CDCl_3_) δ: 11.9, 26.1, 27.1, 29.6, 30.1, 31.7, 34.1, 34.3, 36.7, 38.4, 43.0, 44.1, 44.2, 48.4, 88.0, 116.7, 117.1, 119.5, 121.4, 122.0, 126.8, 129.9, 135.0, 136.1, 138.7, 154.1, 158.3, 171.3. HRMS for C_28_H_35_Br^81^NO_3_ [M+H]^+^: Calculated 514.1779 and found 514.1772 *m/z*. HPLC purity: 83.6% (RT = 20.4 min). (Supplementary Materials).

### 2.3. Biology

#### 2.3.1. Cells for Enzymatic and Proliferative Assays

*T-47D cells*: Human T-47D breast cancer cells were obtained from American Type Culture Collection (Manassas, VA, USA) and cultured in RPMI-1640 supplemented with L-glutamine (2 nM), penicillin (100 IU/mL), streptomycin (100 µg/mL), 17β-estradiol (1 nM) and 10% (*v/v*) fetal bovine serum (FBS). Cells were maintained under a 5% CO_2_ humidified atmosphere at 37 °C. The culture media were changed every two or three days and the cells were split once a week to maintain cell propagation.

*Z-12 cells*: Human Z-12 endometriotic epithelial cells were obtained from Dr. Asgi T. Fazleabas (Michigan State University, Grand Rapids, MI). Cells were grown in Dulbecco’s Modified Eagle medium (DMEM/F12) supplemented with 5% (*v/v*) FBS treated with dextran-coated charcoal, L-glutamine (2 nM), penicillin (100 IU/mL) and streptomycin (100 µg/mL). Cells were maintained under a 5% CO_2_ humidified atmosphere at 37 °C. The culture media were changed every two or three days and the cells were split once a week to maintain cell propagation.

#### 2.3.2. 17β-HSD1 Inhibition Assay

T-47D cells were grown in a medium supplemented with insulin (50 ng/mL) and 5% dextran-coated charcoal-treated FBS, which was used rather than untreated 10% FBS, to remove the remaining steroid hormones. Stock solution of each compound to be tested was previously prepared in DMSO and diluted with a culture medium to achieve the appropriate concentrations, prior to use. For the assay, the cells (8000 cells/well) were seeded in a 24-well plate. After 24 h of incubation, a diluted solution of each inhibitor was added to the cells to obtain the appropriate final concentration (0.1, 1 and 10 μM). The final concentration of DMSO in the well was adjusted to 0.03%. The inhibitor and cells were preincubated for 1 h at 37 °C and a solution containing [^3^H]-E1 (7 nM) and untritiated E1 (53 nM) was added to obtain a final concentration of 60 nM. Cells were incubated for 24 h and each inhibitor was assessed in triplicate. After incubation, the culture medium was removed, and the steroids (tritiated and untritiated E1 and E2) were extracted with diethyl ether. The organic phase was evaporated to dryness with nitrogen. Residues were dissolved in CH_2_Cl_2_, spotted on silica gel TLC plates (EMD Chemicals Inc., Gibbstown, NJ, USA) and eluted with toluene/acetone (4:1) as the solvent system. Substrate [^3^H]-E1 and metabolite [^3^H]-E2 were identified by comparison with reference steroids (E1 and E2), and isolated. Each compound was quantified with a scintillation counter. The percentage of transformation and of inhibition were calculated as follows: % transformation = 100 [^3^H]-E2 / ([^3^H]-E1 + [3H]-E2) and % inhibition = 100 (% transformation without inhibitor − % transformation with inhibitor)/% transformation without inhibitor. The concentration inhibiting 50% of E1 to E2 transformation (IC_50_) was determined using GraphPad Prism 6 software.

Z-12 cells were tested using the same protocol reported above for T-47D cells except for the cell medium, the cell number (100,000 to 750,000 cells/well) and the incubation time (24, 48 and 72 h).

#### 2.3.3. Cell Viability Assay

The cell proliferation assays were performed using 3-(4,5-dimethylthiazol-2-yl)-5-(3-carboxymethoxyphenyl)-2-(4-sulfophenyl)-2H-tetrazolium (MTS) (Cell Titer 96 Aqueous, Promega, Nepean, ON, Canada), as previously described [28,29]. Briefly, cells (5000/well for T-47D or 3000/well for Z-12) were plated in triplicate in 96-well plates in appropriate culture medium (total of 90 µL). Before each treatment, the cells were incubated at 37 °C in a 5% CO_2_ humidified atmosphere for 24 h. Compounds **5**, **6**, **10** and RM-581, as positive control [30,31], were dissolved in DMSO to obtain stock solutions, which were diluted at multiple concentrations with culture media to obtain the desired final concentration by adding 10 µL in each well, and the mixture was incubated for 3 days. Following treatment, 20 µL of a solution of MTS were added to each well and the mixture was incubated for 4 h. The plates were subsequently analyzed at 490 nm using a Tecan M-200 microplate reader (Männedorf, Switzerland) and the concentration inhibiting 50% of cell proliferation was determined using GraphPad Prism 6 software.

### 2.4. Molecular Modeling

A docking study was performed using GOLD software version 5.5 (The Cambridge Crystallographic Data Centre, Cambridge, UK). The X-ray structure of 17β-HSD1 in complex with estrone was retrieved from Protein Data Bank entries 6MNE [19,32]. Ligands (inhibitors **5**, **6**, **10** and EM-251) were energy-minimized using ChemBio3D version 13.0 software (CambridgeSoft, PerkinElmer). Using the GOLD wizard, the proteins were prepared by adding hydrogens, deleting water molecules, and extracting the co-crystallized ligands. The 17β-HSD1 ligands were docked at the binding site within an 8 Å radius sphere using the following parameters: 100 genetic algorithm (GA) runs and 125,000 operations. ChemPLP fitness function was chosen as scoring function, within the Gold_nuclear_hormone_rec_VS template. The dockings were ranked according to the value of the ChemPLP fitness function; only the best ranked solutions data were selected.

Docking experiments were carried out using the wizard function, applying the following sequence of operations: (1) Protonation of the protein by adding hydrogens; (2) deletion of water molecules; (3) extraction of the ligand E1; (4) definition of the active site with a 8 Å radius sphere by selecting the active site residue of protein; (5) selection of the options related to detect cavity atoms file from the selection; (6) selection of the option to force all H-bond donors/acceptors to solvent-accessible surface; (7) loading of the configuration template for Gold_nuclear_hormone_rec_VS template; (8) addition of the minimized ligand, which was minimized with ChemBio3D version 13.0 software and save as sdf file; (9) use of the GA settings for all calculations and a set of 100 solutions were saved for each ligand; (10) use of the ChemPLP fitness function as scoring function; (11) setting of the genetic algorithm option to slow (most accurate) mode; and (12) running Gold.

## 3. Results and Discussion

### 3.1. Chemical Synthesis

**Scheme 1:**
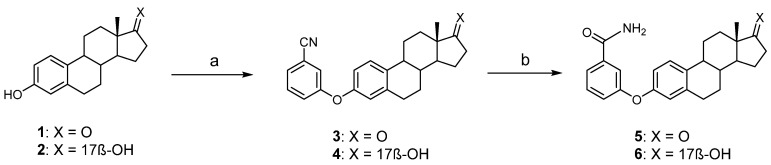
Chemical synthesis of compounds **5** and **6**. Reagents and conditions: (**a**) 3-Fluorobenzonitrile, K_2_CO_3_, DMSO, MW 300 W, 190 °C, 1 min. (**b**) Acetamide, PdCl_2,_ THF/H_2_O, rt, 24 h.

**Scheme 2:**
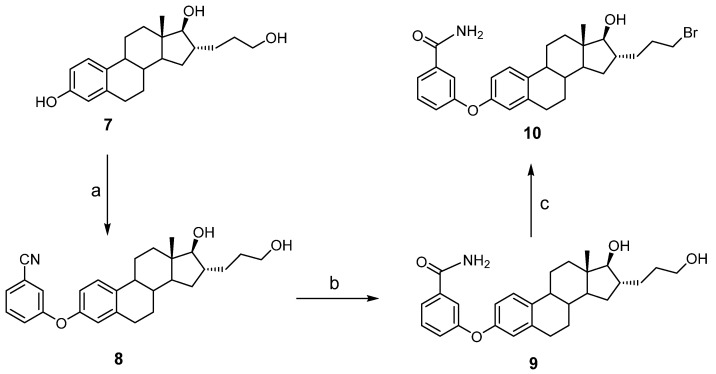
Chemical synthesis of compound **10**. Reagents and conditions: (**a**) 3-Fluorobenzonitrile, K_2_CO_3_, DMSO, MW 300 W, 190 °C, 1 min. (**b**) Acetamide, PdCl_2_, THF/H_2_O, rt, 24 h. (**c**) PPh_3_, TBAI, dibromoethane, 60 °C, 24 h.

The synthesis of compounds **5** and **6** was carried out in two steps starting from estrone (E1) and estradiol (E2), respectively (Scheme 1 and Scheme 2). For the first step, we used the conditions described by Li et al. [33] for the microwave-assisted synthesis of diaryl ethers. Using E2, we attempted to optimize the yield of this reaction to obtain intermediate **4**, but the optimal conditions did not allow the reaction to be complete. Indeed, by heating at 100 °C and 150 °C for 5 min, the reaction was only 5% and 20% completed. By increasing the temperature to 170 °C, 60% and 51% yields were obtained after 3 and 7 min, respectively. In the latter case, however, the appearance of a slightly less polar by-product was observed by TLC, while extending the heating time to 10 min did not improve the yield. Thus, the parameters to obtain maximum expected product were heating at 190 °C for 1 min.

Using the conditions optimized for the formation of diaryl ether **4**, 3-fluorobenzonitrile reacted with the phenol at position 3 of steroids **1** and **2** to yield compounds **3** and **4** in 30% and 71%, respectively, after purification by silica gel chromatography. Purification was difficult, due to the presence of a by-product with a retention time almost like that of compound **3**, which explains the low yield of this ether compound. The substitution of 3-fluorobenzonitrile to produce ethers **3** and **4** was confirmed in IR spectroscopy by the disappearance of the band characteristic of the starting phenol (E1) as well as the appearance of a band of weak intensity but very characteristic for the presence of a nitrile group (2230 cm^−1^). In ^1^H NMR spectrum, we also observe the appearance of signals at 7.16, 7.32, 7.40 and 7.46 ppm corresponding to the aromatic protons of the benzonitrile nucleus. In addition, a deshielding effect of the aromatic protons corresponding to positions 1, 2 and 4 of the steroid A-ring was observed compared to the same signals in the starting compounds **1** (E1) and **2** (E2). As examples, 7.16, 6.80 and 6.73 ppm were obtained for H1, H2, and H4 of compound **3** and 7.07, 6.52 and 6.45 ppm for starting compound E1. In ^13^C NMR, the presence in the aromatic region of 13 peaks instead of 6 peaks for E1 and E2 supports the substitution of 3-fluorobenzonitrile by the phenol of E1 and E2 to generate ethers **3** and **4**, respectively.

After obtaining compounds **3** and **4**, the next step consisted of transforming the nitrile into a carboxamide group in the presence of acetamide, water and PdCl_2_ catalyst to obtain corresponding carboxamides **5** and **6** with excellent yields of 91% and 89%, respectively, after purification by chromatography. The metal catalyzed transfer hydration of nitrile to amide was supported with the IR spectra by the disappearance of the nitrile band and the appearance of an amide band (1659 cm^−1^). In addition, the presence of two large singlets (5.59 and 6.03 ppm for **5** and 5.91 and 6.09 ppm for **6**) in ^1^H NMR and a signal at 169.3 ppm in ^13^C NMR are characteristic of the CONH_2_ group.

Compound **10** was obtained in three steps from alcohol **7** [26] (Scheme 2). The first two steps, similar to those described in order to obtain ethers **3** and **5**, made it possible to obtain intermediate products **8** and **9** with yields of 70% and 67%, respectively. For the third step, the bromination of alcohol **9** to generate **10**, it was not possible to use the classical Appel bromination conditions (triphenylphosphine (PPh_3_) and CBr_4_ in dichloromethane) because they lead to the transformation of the carboxamide group of **9** to the corresponding nitrile **8**. To avoid this unwanted reaction, we alternatively used a method initially developed by Chen et al. [34] using PPh_3_, tetrabutylammonium iodide (TBAI) and dibromoethane. Under the conditions deemed optimal for the bromination of a primary alcohol in the presence of a *m*-carbamoyl phenyl group [35], compound **10** was however obtained with a low yield of 26%. An incomplete reaction and a difficult purification by silica gel chromatography explain this low yield. The formation of the desired compound **10** was confirmed by the signal at 3.45 ppm (C**H**_2_Br) in ^1^H NMR while the signal at 34.2 ppm in ^13^C NMR is in agreement with the literature data for a CH_2_Br on an alkyl chain [26,27]. The two peaks of the same intensity appearing on the mass spectrum are also characteristic of the presence of a bromine atom and of the substitution of the primary alcohol.

### 3.2. Inhibition of 17β-HSD1 Activity Transforming E1 into E2

T-47D breast cancer cells are known to efficiently convert E1 into E2 [36]. In fact, we previously demonstrated that the relatively low level of mRNA expression of 17β-HSD1 in these cells is responsible for the high transformation rate of E1 into E2, compared to other reductive 17β-HSDs (types 7 and 12) [36]. For this reason, T-47D cells are a very good cell model to test the inhibitory activity of molecules synthesized as potential inhibitors. In our experiments, whole T-47D cells containing a mixture of E1 and [^3^H]-E1 (total of 60 nM) were incubated for 24 h in the presence or not of different compounds and the % of transformation ([^3^H]-E1 to [^3^H]-E2) was calculated and next the % of inhibition was calculated (Table 1).

At concentrations of 0.1, 1 and 10 µM, the reference inhibitor PBRM [23,35] inhibited 44, 75 and 91% of 17β-HSD1 activity (IC_50_ = 0.15 µM) while the unlabeled natural substrate E1 produced 45, 61 and 78% of inhibition (IC_50_ = 0.21 µM). The addition of the *m*-carbamoylphenyl group at C3 of E1 with the formation of an ether bond (compound **5**) slightly reduced the inhibitory activity (IC_50_ = 0.21 and 0.31 μM for E1 and **5**, respectively). The inhibitory activity of compound **5** (IC_50_ = 0.31 μM) is, however, close to that of the PBRM inhibitor (IC_50_ = 0.15 μM), a targeted-covalent inhibitor which has demonstrated its potential during proof of concept for the treatment of breast cancer [37] and endometriosis [38]. The inhibitory capacity of E1-derivative **5,** however, decreased when the ketone at C17 was reduced into 17β-alcohol (E2-derivative **6**) (IC_50_ = 0.31 and 1.2 μM, respectively). Since the addition of a *m*-carbamoylphenyl group at C3 was reported to favor the inhibition of 17β-HSD1 [19], we also tested this approach on a known 17β-HSD1 inhibitor, the E2 derivative EM-251 [26,27]. Unfortunately, the inhibitory activities of compound **10**, a hybrid molecule having an ether group at C3 and a bromopropyl chain at C16α, was 2-fold lower (IC_50_ = 1.3 μM) than the IC_50_ (0.58 μM) of EM-251.

Z-12 endometriosis cells were also used as a potential source of 17β-HSD1 activity converting E1 into E2 (Figure 1). We tested different cell numbers (100, 200, 500 and 750 × 10^3^) and different incubation times (24, 48 and 72 h), but the E1 into E2 conversion rates were very low, variable and less than 7%. For example, only 4% transformation was observed, despite the use of 200 × 10^3^ Z-12 cells and an incubation time of 24 h. Thus, although Z-12 cells express the 17β-HSD1 mRNA [39,40], this does not seem to translate into E2 formation. Intrigued by this surprising result, we also tested the activity of aromatase, another steroidogenesis enzyme whose mRNA is expressed in Z-12 cells [39,40,41]. By incubating 5, 10, 25, 50, 100, 300 and 600 × 10^3^ Z-12 cells in the presence of [1β-^3^H]-4-androstene-3,17-dione (60 nM) for 1, 4 and 24 h, we did not observe a significant release of tritiated water, which would be representative of the aromatization of the A-ring of the natural aromatase substrate 4-DIONE to form E1 (Figure 1). In summary, despite expressing 17β-HSD1 and aromatase, Z-12 cells do not appear to be able to produce significant amounts of E2 and E1, respectively. Z-12 cells would therefore not be an adequate model for testing potential inhibitors of aromatase and 17β-HSD1, two enzymes responsible for estrogen production.

### 3.3. Molecular Docking

To better visualize the molecular interactions between 17β-HSD1 and compounds **5**, **6** and **10**, we performed molecular docking experiments with GOLD software. The docking was built from a PDB ID 6MNE file [19,32], which is a co-crystallized structure of E1 and 17β-HSD1. This co-crystal structure is particularly interesting, since the position of estrone in the catalytic site of 17β-HSD1 is in reverse to what is classically admitted [11,20]. Indeed, the 17-ketone is close to His-221 and far away from the well-know catalytic triad amino acids (Ser-142, Tyr-155 and Lys-159) involved in hydride transfer from co-factor toward the stereoselective reduction of the 17-ketone to the corresponding 17β-OH of E2 [11]. On the other side, the phenolic OH at position C3 points to the catalytic amino acid triad region rather than to His-221, contrary to what is observed for E2 (PDB ID 1IOL) [42] where phenol is close to His-221 [43].

Recently, Li et al. [19] proposed that the addition of a *m*-carbamoylphenyl group at C3 of E1 (compound **5**) would be a potential inhibitor of 17β-HSD1. In fact, their preliminary docking experiments suggested that the steroidal scaffold would be well tolerated in the reverse orientation, as observed for E1 by moving the C17-carbonyl in the region near His-221 and the 3-*m*-carbamoylphenyl moiety close to amino acids of the catalytic triad [19]. Despite the interesting aspect of this hypothesis, the inhibition potential of such a compound was not yet validated experimentally in an enzyme assay. In that mindset, we performed an inhibition assay on 17β-HSD1 with compound **5** (17-C=O) and **6** (17β-OH) to see if those compounds could be valuable inhibitors.

**Figure 4:**
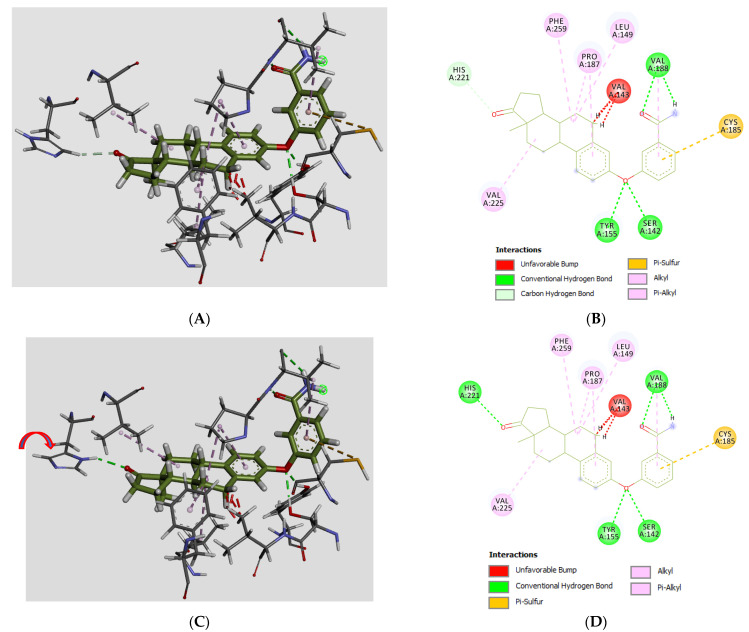
(**A**) The best solution pose (ChemPLP score = 71.2) among 100 solutions generated for compound **5** showing key interactions with amino acids of the catalytic site of 17β-HSD1; (**B**) 2D-interaction diagram showing interaction types in play; (**C**) Imidazole ring of His-221 pivoted by 180 degrees from its original position; (**D**) 2D-interaction diagram showing interaction types in play.

**Figure 5:**
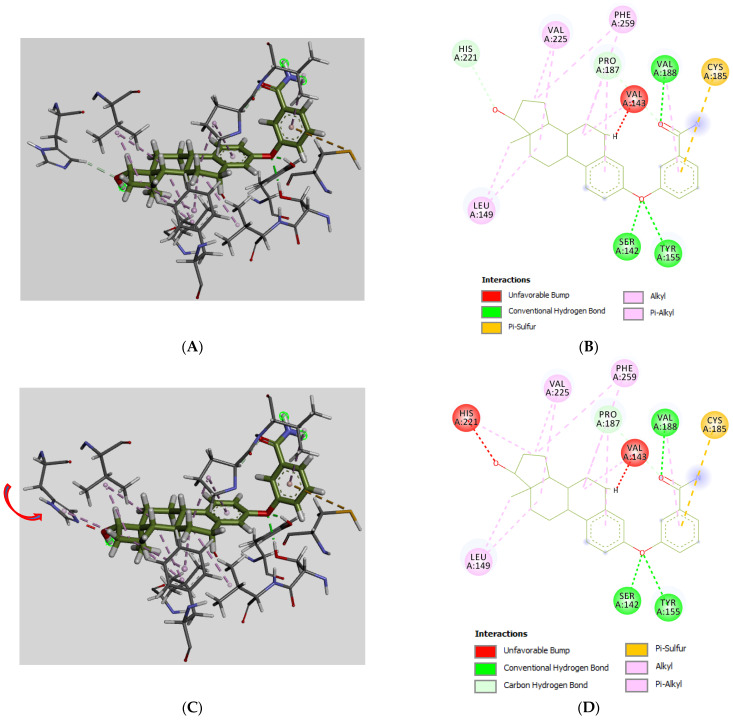
(**A**) The second-best solution pose (ChemPLP score = 69.9) among 100 solutions generated for compound **6** showing key interactions with amino acids of the catalytic site of 17β-HSD1; (**B**) 2D interaction diagram showing interaction types in play; (**C**) Imidazole ring of His-221 rotated from its original position showing a close contact with oxygen of 17β-OH; (**D**) 2D-interaction diagram showing interaction types in play.

Molecular modeling experiments for compounds **5** and **6** were thus performed and some interesting observations emerged (Figure 4 and Figure 5). First, in the case of 3-*m*-carbamoylphenyl-E1 (**5**), the best solution poses obtained were in the reverse-position orientation, as previously suggested by Li et al. [19]. Notably, among the 10 best solution poses (highest ChemPLP scores) obtained over the 100 solutions generated during the docking experiment, nine of them were in the reverse position and only one was in the classical position (normal orientation). Interestingly, for the best pose solution (ChemPLP score = 71.2) four hydrogen bonds take place over the 3-*m*-carbamoylphenyloxy moiety (Figure 4A,B), including two H-bonds between Tyr-155/Ser-142 and the two lone pairs of the diaryl ether, as well as two other H-bonds between Val-188 and the carbonyl and the N-H of the carboxamide group (CONH_2_). Notably, a fifth H-bond between N-H of His-221 and the carbonyl group of C17-ketone was also obtained when the imidazole ring of His-221 was flipped by 180 degrees (Figure 4C). Otherwise, a *pi* sulfur interaction between Cys-185 and the aromatic ring of *m*-carbamoylphenyloxy moiety is present, as well as alkyl-alkyl and *pi*-alkyl interactions with the steroidal scaffold, which adds to the favorable H-bond interactions. Finally, an unfavorable steric interaction between Val-143 and the C6 methylene group of the steroidal scaffold was observed.

In the case of the 3-*m*-carbamoylphenyl-E2 (**6**), the second-best solution pose (ChemPLP score = 69.9) is depicted in Figure 5A and the *m*-carbamoylphenyloxy moiety shows the same interactions as for compound **5** (Figure 5B). However, no H-bond between 17β-OH and His-221 occurs, but a weaker carbon hydrogen bond interaction takes place between the C-H of imidazole ring and the oxygen lone pair of 17β-OH. Furthermore, even if the His-221 imidazole ring was turned around its axis, it remains impossible to reach an optimal angle to favor the formation of an H-bond. In fact, in that process, an unfavorable steric clash with the oxygen of the 17β-OH group was obtained when the His-221 imidazole ring is at its closest position to the hydrogen of 17β-OH (Figure 5C,D). Otherwise, in a similar manner as compound **5**, less energetic interactions (alky-alkyl, *pi*-alkyl, *pi*-Sigma) than H-bond took place between Val-225, Phe-259, Pro-187 and Leu-149 and the steroidal scaffold, as well as an unfavorable steric interaction between Val-143 and the C6-methylene group. Finally, IC_50_ values of compounds **5** and **6** (IC_50_ = 0.31 and 1.2 µM, respectively) support the fact that an additional H-bond at position C-17 for compound **5** seems to be an important factor contributing to the overall better compound affinity for 17β-HSD1, and consequently to an increased inhibition activity of **5** vs. **6**.

**Figure 6:**
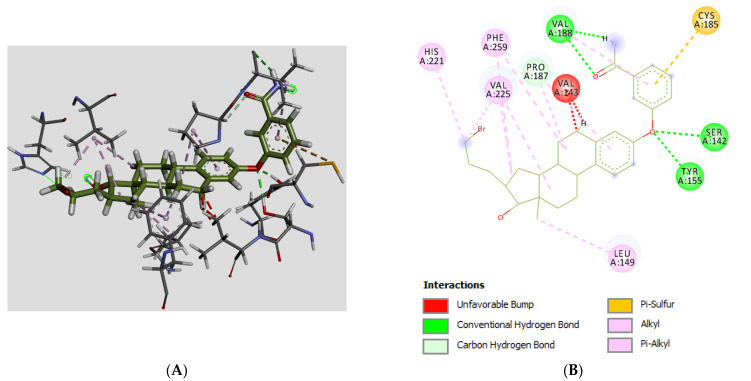

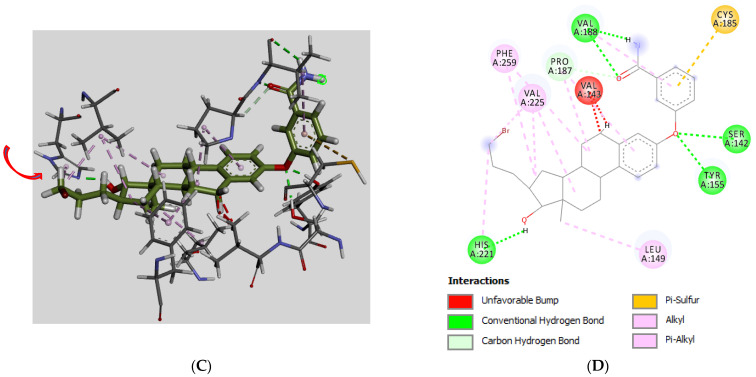
(**A**) The best solution pose (ChemPLP score = 73.3) among 100 solutions generated for compound **10** showing key interactions with amino acids of the catalytic site of 17β-HSD1; (**B**) 2D-interaction diagram showing types of interaction in play; (**C**) Imidazole ring of His-221 moved from its original position leading to the formation of a H-bond with 17β-OH; (**D**) 2D-interaction diagram showing types of interaction in play.

**Figure 7:**
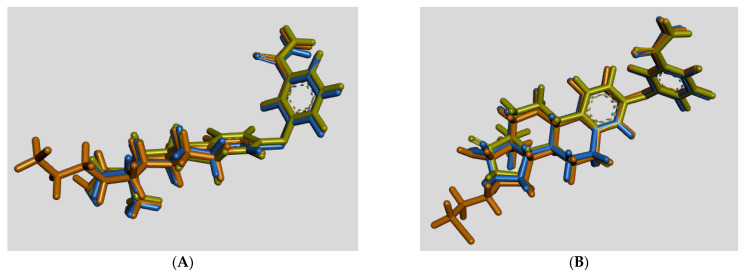
Superimposition of best solution poses obtained for compounds **5** (in yellow), **6** (in blue) and **10** (in orange) showing a strong overlap of the three molecules. (**A**) side view; (**B**) top view.

Knowing that the replacement of the phenol of reversible 17β-HSD1 inhibitor CC-156 [24] by a small bromoethyl chain (inhibitor PBRM) leads to the formation of a covalent bonding with His-221 and an irreversible inhibition [23,44], we seized the opportunity to introduce a bromopropyl chain at position 16 of compound **6** (leading to compound **10**, a C3-ether version of the known inhibitor EM-251 [26,27,28]). In fact, we considered that the proximity of the side-chain’s CH_2_Br to His-221 might favor the formation of a covalent bond. In that mindset, and as previously performed for compounds **5** and **6**, we performed a molecular docking experiment analysis for compound **10**. Interestingly, the best solution (ChemPLP score = 73.3) obtained among the 100 solutions generated shows that interactions with the 3-*m*-carbamoylphenyloxy moiety are the same as those found for compounds **5** and **6** (two H-bond with of the diarylether, two H-bonds with the carboxamide group, and *pi*-sulfur with an aromatic ring (Figure 6A,B). However, contrary to compound **5** and as seen for compound **6**, no H-bond interactions were seen with the 17β-OH. No extra interactions were observed with the methylene of the bromopropyl chain. These observations are in agreement with the measured IC_50_ (1.3 µM) of compound **10**, which was higher than IC_50_ (0.31 µM) of compound **5** and in the same range as the IC_50_ value of compound **6** (1.2 µM), also missing an additional H-bond with His-221. We also expected that if a covalent bonding occurred, as observed for the PBRM inhibitor [44], a stronger inhibition activity would have been measured, but that was not the case. In addition, the distance of the side chain CH_2_Br group from His-221 (3.6 Å) seems to be a little bit too far to favor the formation of a strong covalent bond. Finally, even if the formation of an H-bond between a nitrogen atom of imidazole ring and the hydrogen of 17β-OH was found (possible by rotating His-221 (Figure 6C,D)), by doing so, we simultaneously disqualified the availability of nitrogen lone pair toward a nucleophilic attack of the CH_2_Br. Superimposition of the best solution poses of compounds **5**, **6** and **10** showed an almost perfect overlapping between their respective steroidal scaffolds (Figure 7A,B) and provided valuable views to better appreciate the point of differentiation regarding their interaction with the 17β-HSD1 catalytic site.

### 3.4. Antiproliferative Activity

A single proliferation assay on two cell lines (T-47D and Z-12) was used to test compounds **5**, **6** and **10** and thus assess two different characteristics: (1) their potency to reduce the proliferation of estrogen-dependent (ER+) cells and (2) their failure to stimulate these two ER+ cell lines (Table 2). T-47D cells were immortalized from pleural effusion of ductal carcinoma found in the mammary gland of a human patient with breast cancer. They express the estrogen receptor (ER) and 17β-HSD1, the latter enzyme being responsible for the majority of the E2 produced from E1 in this cell line [38]. Z-12 epithelial cells were immortalized from the endometrium of a human patient with endometriosis, and they express ER as well as 17β-HSD1 [40,41]. As discussed above, however, the enzymatic activity transforming E1 into E2 was found to be very low, although the mRNA of 17β-HSD1 was detected in Z-12 cells. We used RM-581 as a reference compound for the proliferative assays, because this aminosteroid is known for its potent cytotoxic effect on several cancer cell lines [31]. In our cell viability assay, RM-581 similarly inhibited the proliferation of T-47D and Z-12 cells with IC_50_ values of 0.61 and 1.5 μM, respectively. In comparison to RM-581, which clearly induced significant toxicity for these two cell lines, synthesized compounds were much less cytotoxic. In T-47D cells, compounds **5**, **6** and **10** weakly and moderately inhibited cell proliferation with IC_50_ values of 49, 37 and 14 μM, respectively, and the presence of the C16-bromopropyl side chain reduced the capacity of these cancer cells to proliferate. In Z-12 cells, the proliferation pattern was roughly the same, with IC_50_ values over 30 μM for compounds **5** and **6**, and an IC_50_ of 20 μM for the C3/C16 hybrid compound **10**. Thus, compounds **5**, **6** and **10** reduced the proliferation of ER+ cells, which is desirable if considering a treatment for breast cancer or endometriosis. In addition, and despite the presence of an estrane-based scaffold, compounds **5**, **6** and **10** did not stimulate ER+ cell proliferation suggesting that they will not produce an estrogenic activity, which would not be desirable for the treatment of breast cancer and endometriosis.

## 4. Conclusions

Three steroid derivatives were synthesized and characterized in order to validate or invalidate the working hypothesis: *m*-carbamoylphenyl group added at C3 of an E1 nucleus favors the formation of an inverse orientation complex with 17β-HSD1, which would allow the generation a new type of inhibitors. The formation of an ether bond at C3 of estrane derivatives could also stabilize the molecule by limiting the metabolism linked to the presence of OH at C3. Two cellular models of cancer (T-47D cells) and endometriosis (Z-12 cells) were therefore used as a source of 17β-HSD1 activity, transforming E1 into potent estrogen E2, but only T-47D cells proved to be suitable to test the inhibitory activity of the synthesized compounds. The use of T-47D cells showed that the addition of the *m*-carbamoylphenyl group at C3 of E1 or at C3 of the known inhibitor EM-251 did not improve their inhibitory activity, but the presence on the steroid scaffold of a C17 ketone was less damaging than that of a 17β-alcohol, as corroborated by molecular docking experiments which identified key interactions with 17β-HSD1. These docking experiments also support the fact that these novel inhibitors may act through an opposite orientation than the classical one of natural enzyme substrate E1 and that they show an excellent superimposition of the three structures (Figure 7). In summary, E1-carbamoylphenyl-ether derivative **5** inhibits 17β-HSD1 more (IC_50_ = 0.31 µM) than E2-carbamoylphenyl-ether derivatives **6** and **10** (IC_50_ = 1.2 and 1.3 µM). Interestingly, compounds **5**, **6** and **10** did not stimulate the proliferation of two estrogen-dependent (ER+) cell lines, but instead they were found able to reduce the proliferation of breast cancer (T-47D) and endometriosis (Z-12) cells.

## Figures and Tables

**Table 1:** Inhibition of 17β-HSD1 by various compounds in T-47D breast cancer cells.

#	C3/C17/C16-Groups	Inhibition (% at 0.1 μM)	Inhibition (% at 1 μM)	Inhibition (% at 10 μM)	Inhibition (IC_50_ in μM)
**5** (ether)	3-R′ / 17-C=O / H	29.1 ± 6.2	70.7 ± 14.1	98.9 ± 2.8	0.31
**6** (ether)	3-R′ / 17β-OH / H	40.0 ± 6.5	48.6 ± 15.7	59.6 ± 1.5	1.2
**10** (ether)	3-R′ / 17β-OH / 16α-(CH_2_)_3_Br	28.2 ± 5.4	37.0 ± 14.5	82.1 ± 5.7	1.3
**E1** (phenol)	OH / C=O / H	44.9 ± 14.7	60.9 ± 8.3	78.1 ± 2.2	0.21
**EM-251** (phenol)	OH / 17β-OH / 16α-(CH_2_)_3_Br	43.8 ± 0.2	36.9 ± 9.3	86.6 ± 8.0	0.58
**PBRM** (bromoethyl)	3-(2′-(CH_2_)_2_Br) / 17β-OH / 16β-R″	43.8 ± 3.4	74.8 ± 3.2	91.0 ± 3.8	0.15

Inhibition of the transformation of [^3^H]-E1/E1 (60 nM) to [^3^H]-E2/E2 by 17β-HSD1 in T-47D cells. EM-251 [26,27] and PBRM [23,35] are two E2 derivatives reported as 17β-HSD1 inhibitors. R′: *m*-NH_2_CO-Phenyloxy; R″: CH_2_Phenyl-*m*-CONH_2_.

**Table 2:** Antiproliferative activity of ether derivatives **5**, **6** and **10** in cell lines representative of breast cancer and endometriosis.

#	C3/C17/C16-Groups	T-47D Cells (Breast Cancer) (IC_50_ in μM)	Z-12 Cells (Endometriosis) (IC_50_ in μM)
**5**	3-R / 17-C=O / H	49	>30
**6**	3-R / 17β-OH / H	37	>30
**10**	3-R / 17β-OH / 16α-(CH_2_)_3_Br	14	20
**RM-581**	**--**	0.61	1.5

Cell proliferation after 3 days expressed as IC_50_ value. RM-581 is a cytotoxic aminosteroid used as a positive control [30,31]. R: *m*-NH_2_CO-Phenyloxy.

## Data Availability

Not applicable.

## Supplementary Materials

The following supporting information can be downloaded at: https://www.mdpi.com/article/10.3390/molecules28020632/s1. ^1^H NMR spectra, ^13^C NMR spectra, HRMS spectra and HPLC chromatograms of compounds **5**, **6** and **10**.

## Sample Availability

Samples of the compounds **5** and **6** are available from the authors.
