# Supplementary material for: Chemical Synthesis and Biological Evaluation of 3-Substituted Estrone/Estradiol Derivatives as 17β-Hydroxysteroid Dehydrogenase Type 1 Inhibitors Acting via a Reverse Orientation of the Natural Substrate Estrone
Source: Molecules. 2023 Jan 7;28(2):632. doi: 10.3390/molecules28020632 (PMC9862175; doi:10.3390/molecules28020632)
Supplement: Supplementary file 1 [file molecules-28-00632-s001.zip › molecules-2104451-supplementary.pdf]

Supplementary Materials

Chemical Synthesis and Biological Evaluation of 3-Substituted Estrone/Estradiol Derivatives as 17β-Hydroxysteroid Dehydrogenase Type 1 Inhibitors Acting via a Reverse Orientation of the Natural Substrate Estrone

Adrien Djiemeny Ngueta [1,2], Jenny Roy [1], René Maltais [1] and Donald Poirier [1,2,*]

[1] Laboratory of Medicinal Chemistry, Endocrinology and Nephrology Unit, CHU de Québec Research Center—Université Laval, Quebec, QC G1V 4G2, Canada; adrien.ngueta-djiemeny.1@ulaval.ca (A.D.N.); jenny.roy@crchudequebec.ulaval.ca (J.R.); rene.maltais@crchudequebec.ulaval.ca (R.M.)

[2] Department of Molecular Medicine, Faculty of Medicine, Université Laval, Quebec, QC G1V 0A6, Canada

[*] Correspondence: donald.poirier@crchudequebec.ulaval.ca

Table of contents:

^{1}H NMR spectra of compound **5**

^{13}C NMR spectra of compound **5**

HRMS spectra of compound **5**

HPLC chromatogram of compound **5**

^{1}H NMR spectra of compound **6**

^{13}C NMR spectra of compound **6**

HRMS spectra of compound **6**

HPLC chromatograms of compound **6**

^{1}H NMR spectra of compound **10**

^{13}C NMR spectra of compound **10**

HRMS spectra of compound **10**

HPLC chromatograms of compound **10**

^{1}H NMR spectra of compound **5**

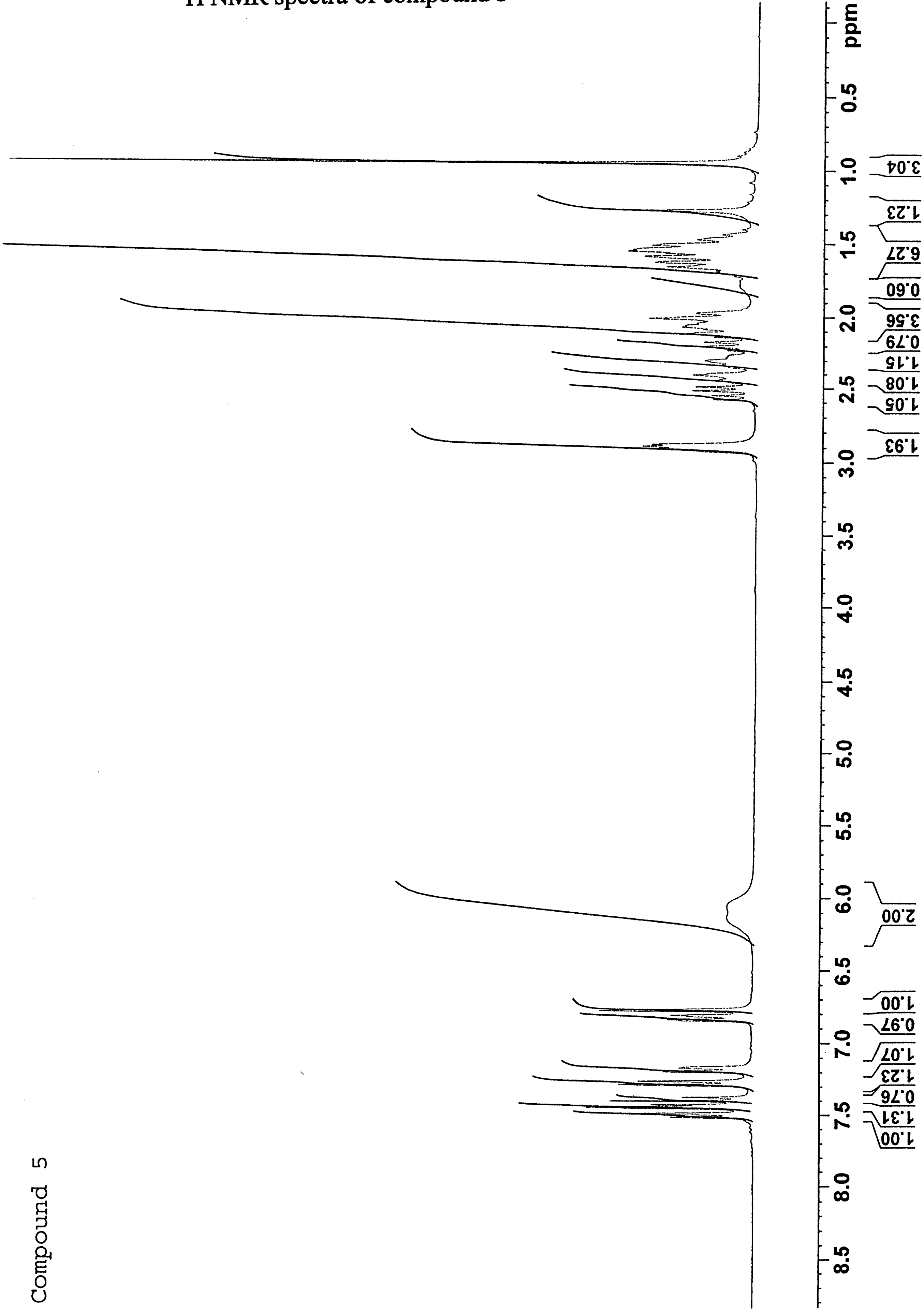

Compound 5

^{13}C NMR spectra of compound **5**

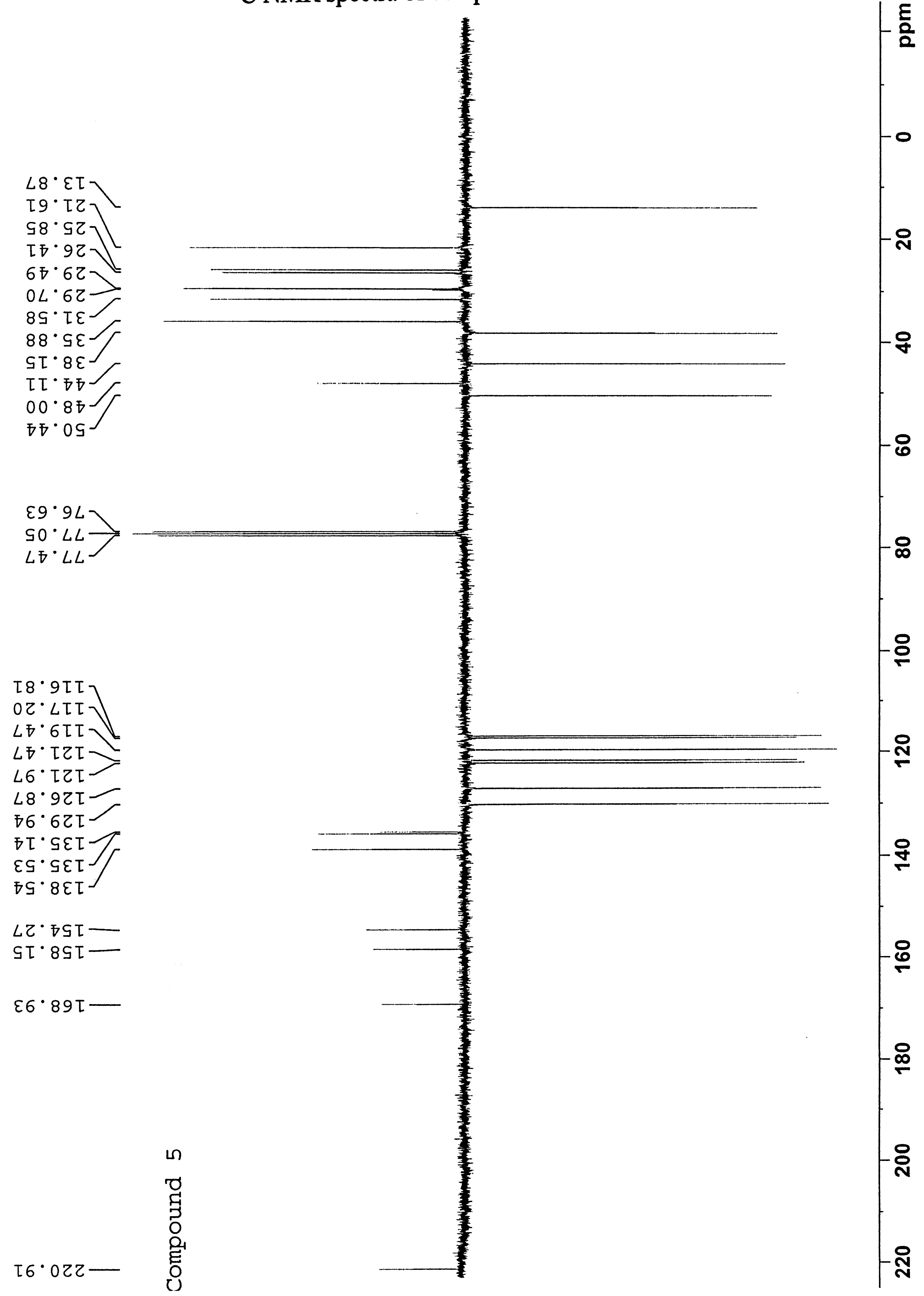

HRMS spectra of compound **5**

Qualitative Compound Report

**Data File**	NGAD-593-144.d	**Sample Name**	NGAD-593-144
**Sample Type**	Sample	**Position**	Vial 66
**Instrument Name**	Instrument 1	**User Name**	Pierre Audet
**Acq Method**	Direct_Injection_ESI.m	**IRM Calibration Status**	[redacted]
**DA Method**	MFC.m	**Comment**	

Compound Table

Compound Label	RT	Mass	Abund	Formula	Tgt Mass	Diff (ppm)
Cpd 1: C25 H27 N O3	0.258	389.2004	2725	C25 H27 N O3	389.1991	3.28

Compound Label	RT	Algorithm	Mass
Cpd 1: C25 H27 N O3	0.258	Find By Formula	389.2004

MS Spectrum

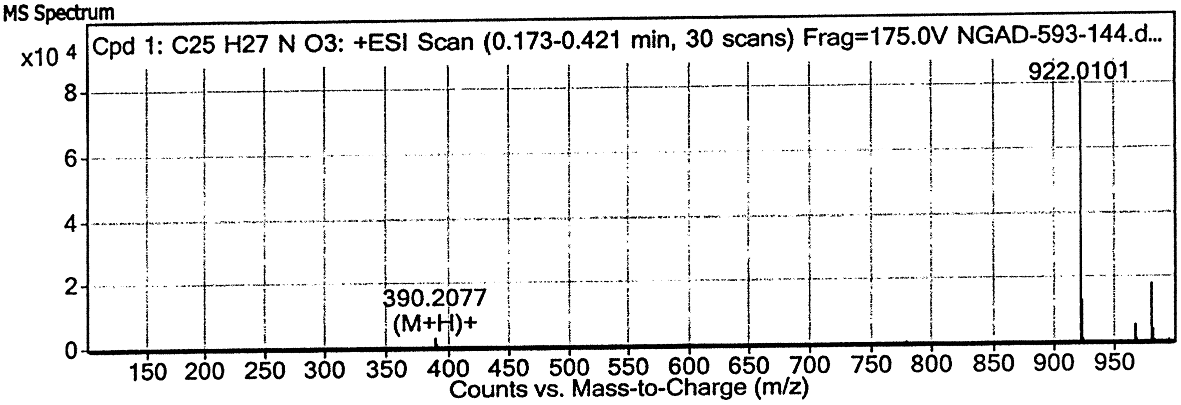

MS Zoomed Spectrum

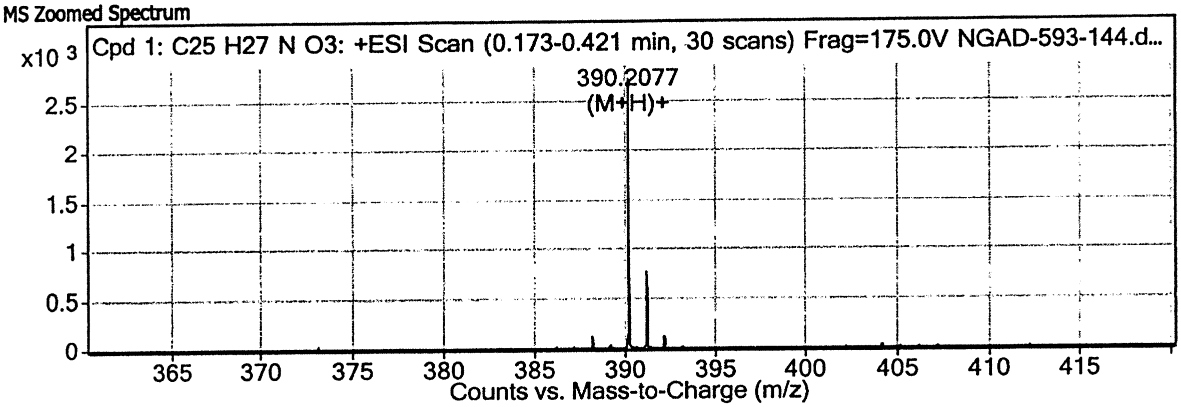

MS Spectrum Peak List

m/z	*Calc m/z*	Diff(ppm)	z	Abund	Formula	Ion
390.2077	390.2064	3.28	1	2745	C25 H28 N O3	(M+H)+
391.2107	391.2097	2.72	1	777	C25 H28 N O3	(M+H)+
922.0101				84640		
922.3857				2036		
922.5534				236		
922.7682				207		
923.0134				12778		
923.3925				281		
924.0163				1612		

HPLC chromatogram of compound 5

==== Shimadzu LabSolutions Analysis Report ====

C:\LabSolutions\Data\Project1\EQUIPE DONALD POIRIER\Adrien NGAD\NGAD-593-146.lcd

Acquired by	: System Administrator
Sample Name	: NGAD-593-144
Sample ID	: NGAD-593-144
Tray#	: 1
Vial#	: 2
Injection Volume	: 8
Data File	: C:\LabSolutions\Data\Project1\EQUIPE DONALD POIRIER\Adrien NGAD\NG
Method File	: C:\LabSolutions\Data\Project1\EQUIPE DONALD POIRIER\Rene Maltais met
Report Format File	: C:\LabSolutions\Data\Project1\EQUIPE DONALD POIRIER\Rene Maltais (div
Month-Day Acquired	: 1/17/2022
Month-Day Processed	: 1/17/2022

<Chromatogram>

C:\LabSolutions\Data\Project1\EQUIPE DONALD POIRIER\Adrien NGAD\NGAD-593-146.lcd

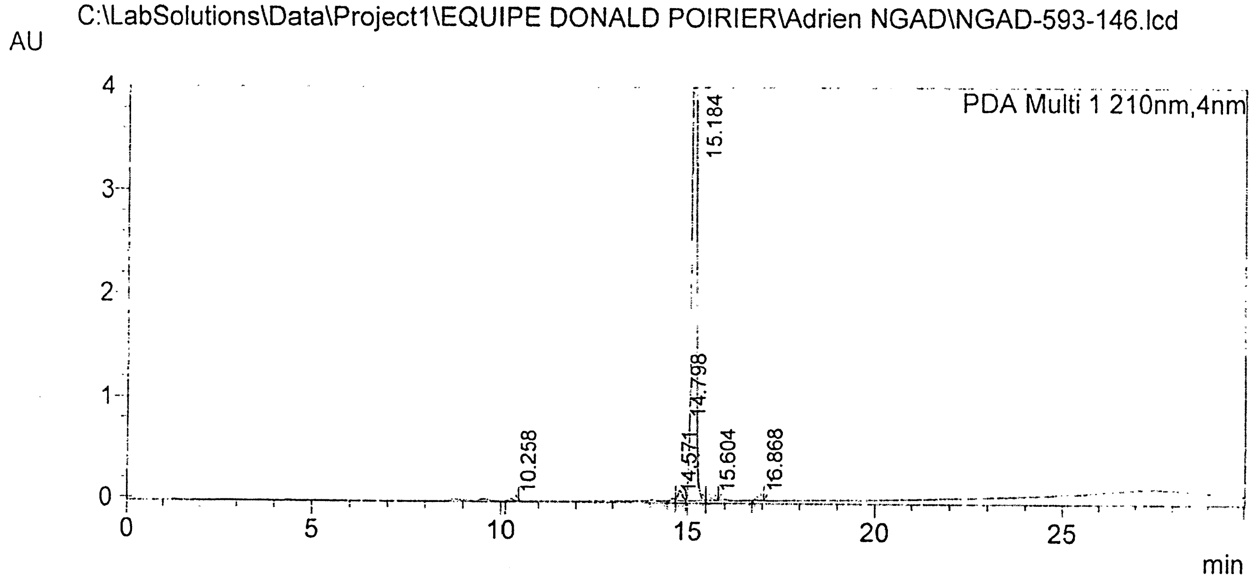

Peak Table

PDA Ch1 210nm

Peak#	Ret. Time	Area	Area%
1	10.258	172237	0.412
2	14.571	147548	0.353
3	14.798	771792	1.846
4	15.184	40252185	96.284
5	15.604	241965	0.579
6	16.868	219818	0.526
Total		41805544	100.000

^{1}H NMR spectra of compound **6**

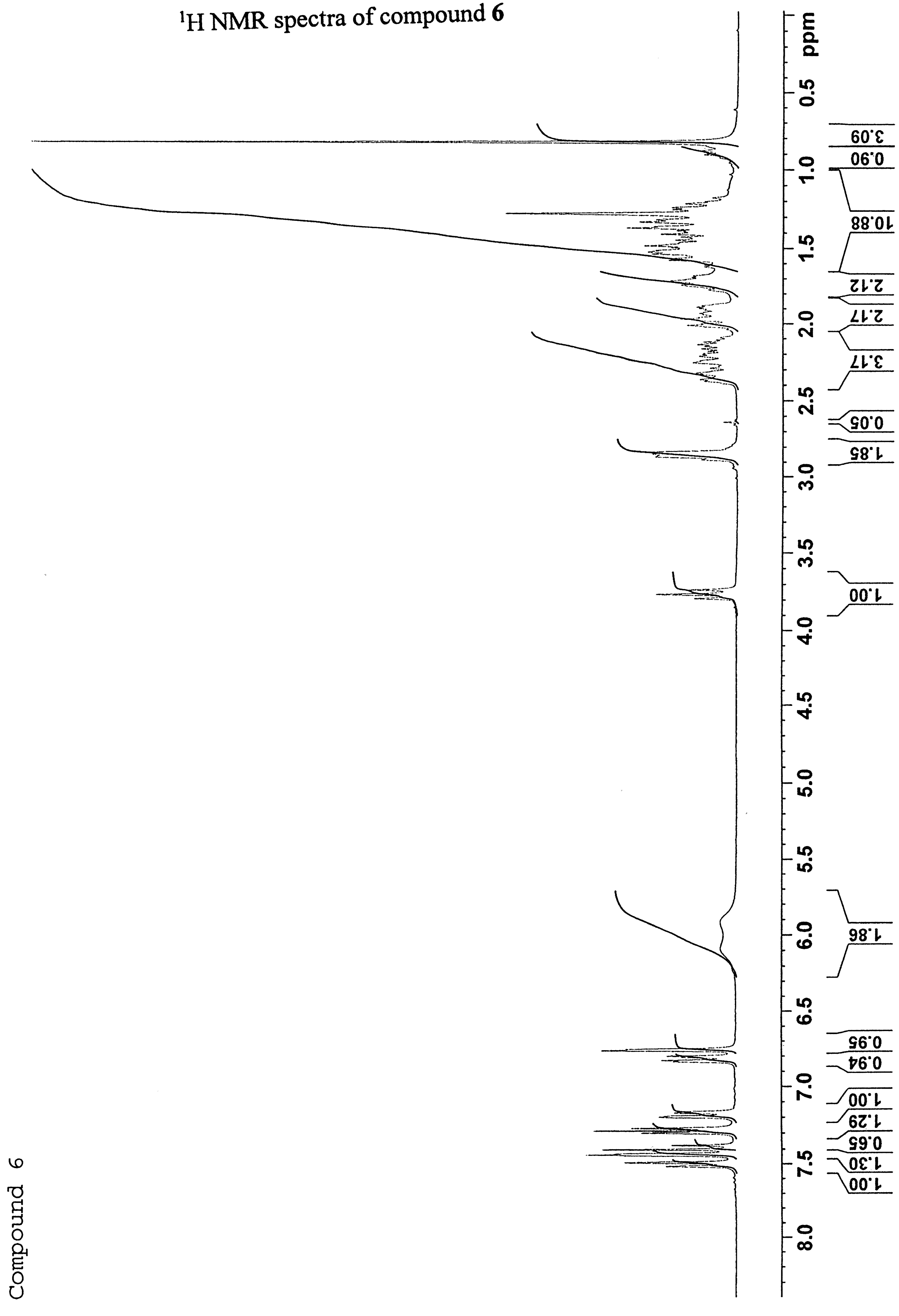

^{13}C NMR spectra of compound **6**

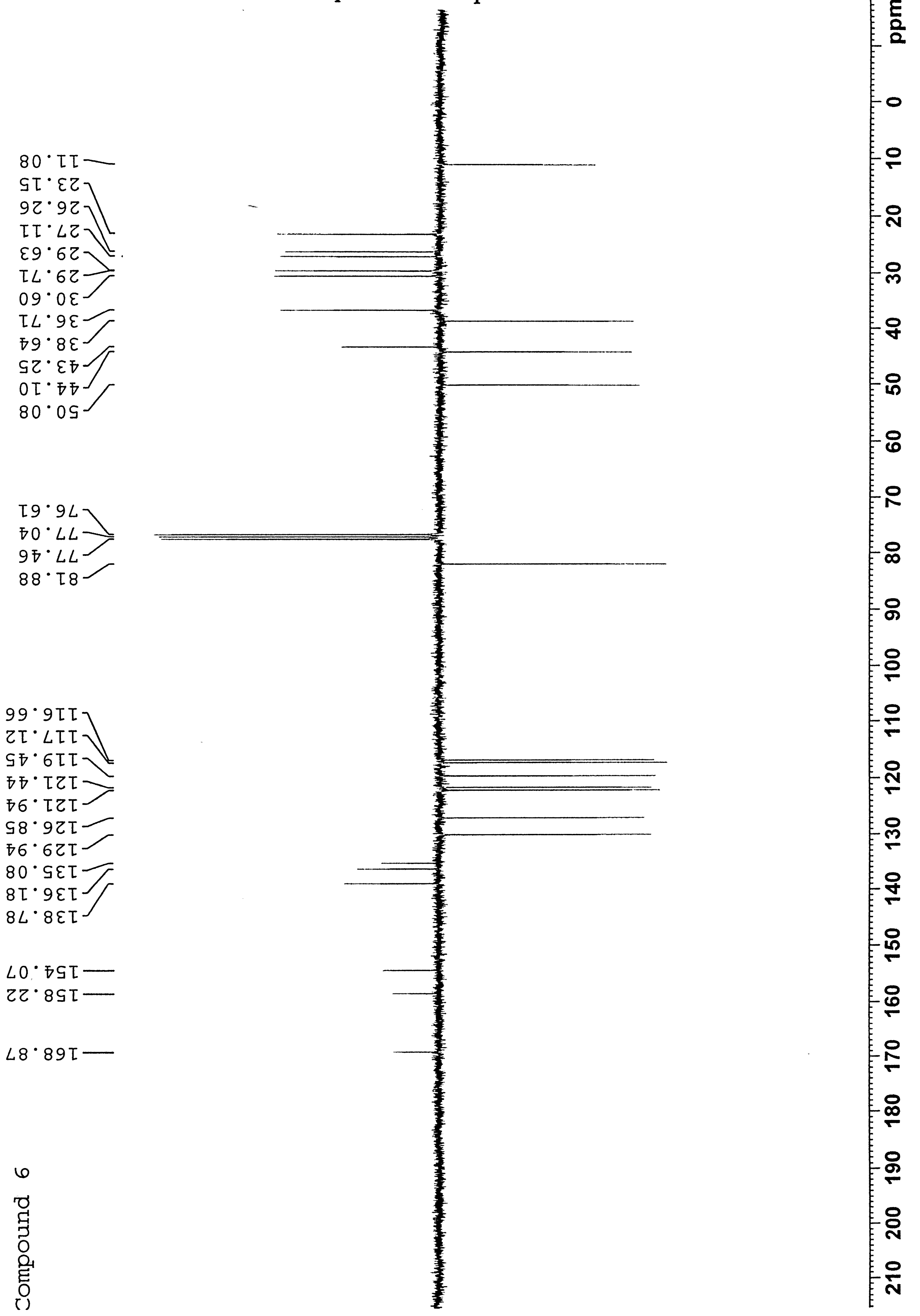

HRMS spectra of compound **6**

Qualitative Compound Report

**Data File**	NGAD-593-142.d	**Sample Name**	NGAD-593-142
**Sample Type**	Sample	**Position**	Vial 65
**Instrument Name**	Instrument 1	**User Name**	Pierre Audet
**Acq Method**	Direct_Injection_ESI.m	**IRM Calibration Status**	[redacted]
**DA Method**	MFC.m	**Comment**	

Compound Table

Compound Label	RT	Mass	Abund	Formula	Tgt Mass	Diff (ppm)
Cpd 1: C25 H29 N O3	0.248	391.2157	4647	C25 H29 N O3	391.2147	2.32

Compound Label	RT	Algorithm	Mass
Cpd 1: C25 H29 N O3	0.248	Find By Formula	391.2157

MS Spectrum

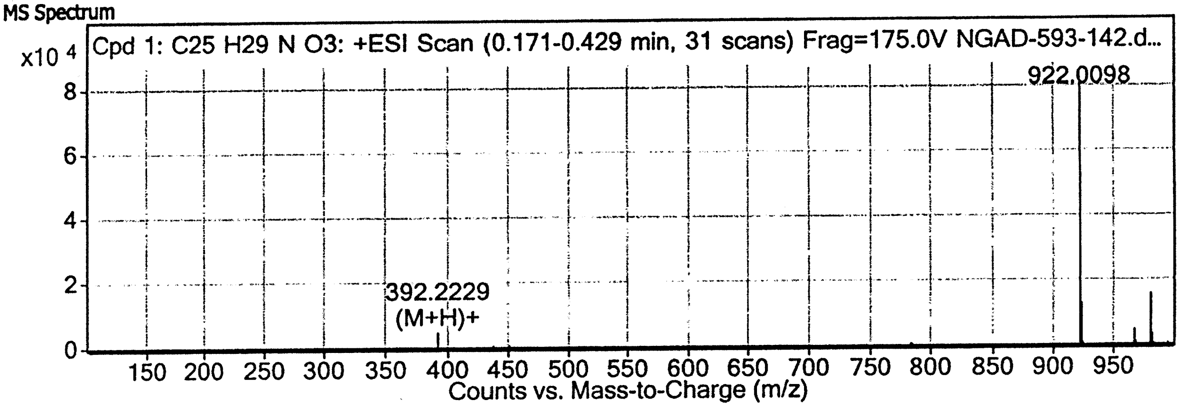

MS Zoomed Spectrum

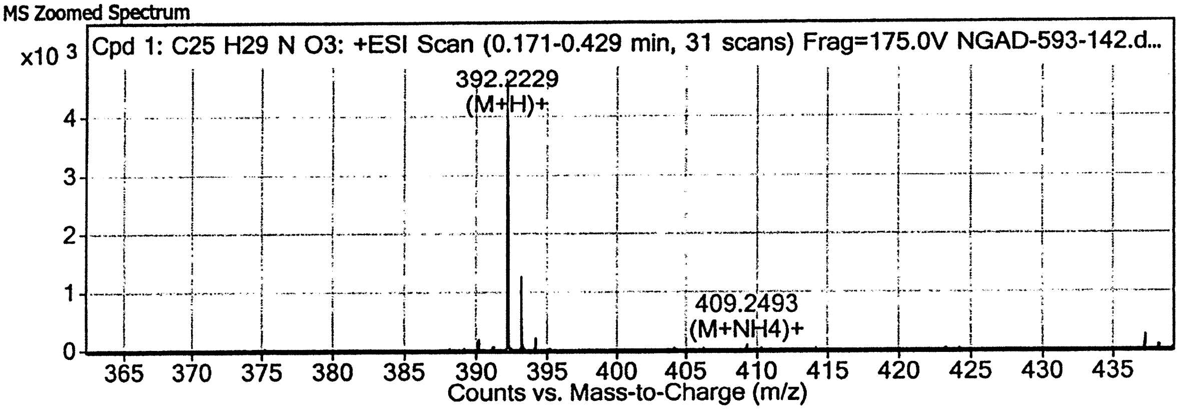

MS Spectrum Peak List

m/z	*Calc m/z*	Diff(ppm)	z	Abund	Formula	Ion
392.2229	392.222	2.32	1	4687	C25 H30 N O3	(M+H)+
393.2262	393.2253	2.29	1	1260	C25 H30 N O3	(M+H)+
409.2493	409.2486	1.9	1	85	C25 H33 N2 O3	(M+NH4)+
922.0098				84125		
922.3853				2018		
922.5563				225		
923.013				12761		
923.3918				274		
924.0156				1570		

HPLC chromatograms of compound 6

==== Shimadzu LabSolutions Analysis Report ====

C:\LabSolutions\Data\Project1\EQUIPE DONALD POIRIER\Adrien NGAD\NGAD-593-143.lcd

Acquired by	: System Administrator
Sample Name	: NGAD-593-142
Sample ID	: NGAD-593-142
Tray#	: 1
Vial#	: 1
Injection Volume	: 8
Data File	: C:\LabSolutions\Data\Project1\EQUIPE DONALD POIRIER\Adrien NGAD\NG
Method File	: C:\LabSolutions\Data\Project1\EQUIPE DONALD POIRIER\Rene Maltais met
Report Format File	: C:\LabSolutions\Data\Project1\EQUIPE DONALD POIRIER\Rene Maltais (div
Month-Day Acquired	: 1/17/2022
Month-Day Processed	: 1/17/2022

<Chromatogram>

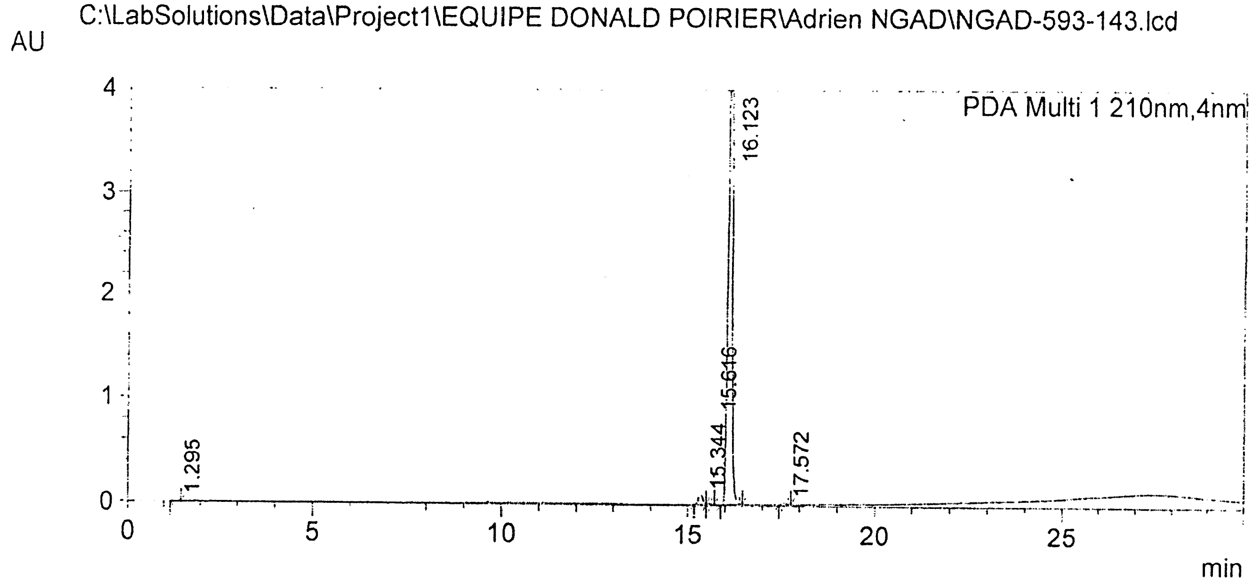

Peak Table

PDA Ch1 210nm

Peak#	Ret. Time	Area	Area%
1	1.295	169349	0.470
2	15.344	650595	1.808
3	15.616	76874	0.214
4	16.123	34956940	97.119
5	17.572	140141	0.389
Total		35993900	100.000

^{1}H NMR spectra of compound **10**

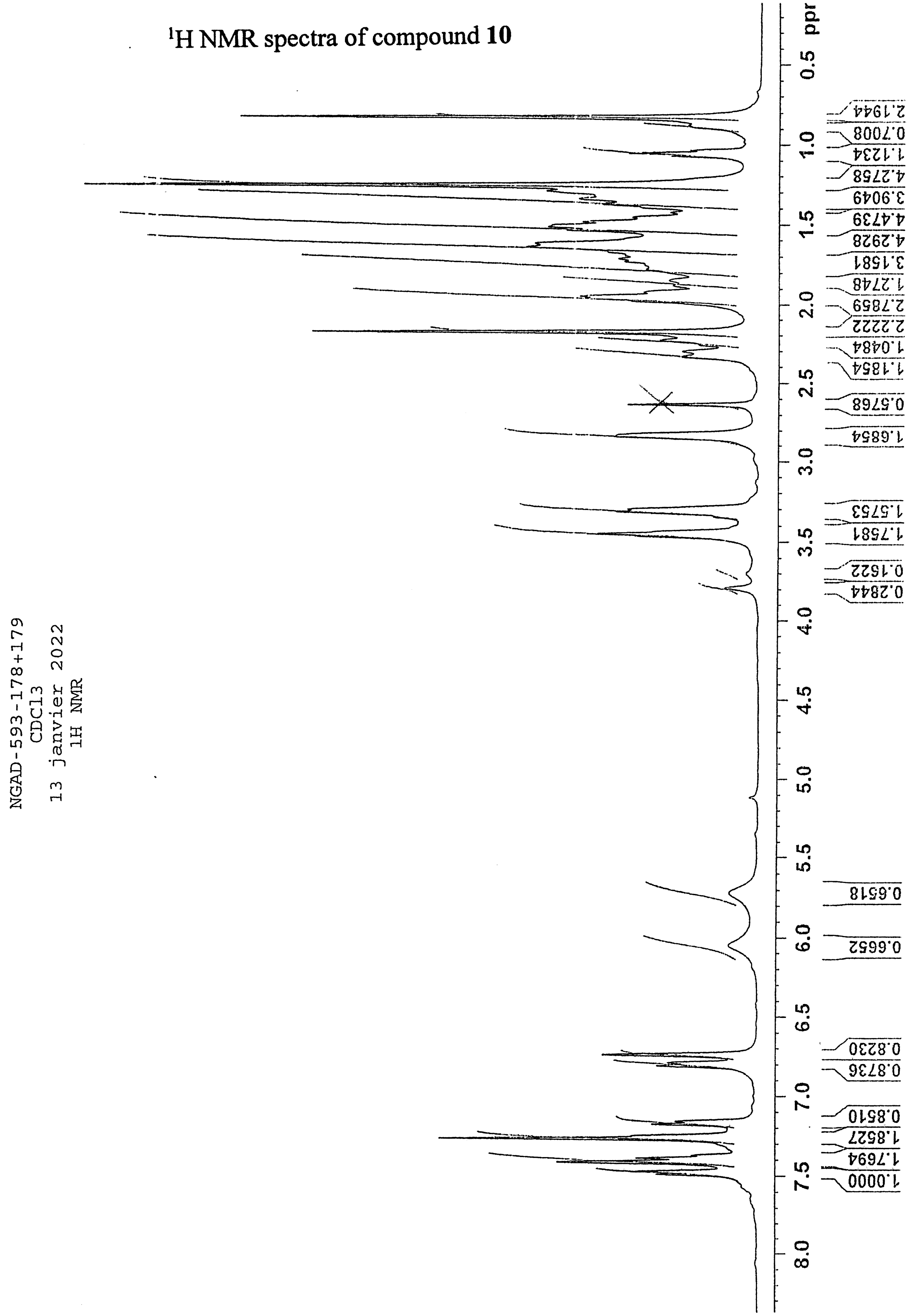

^{13}C NMR spectra of compound **10**

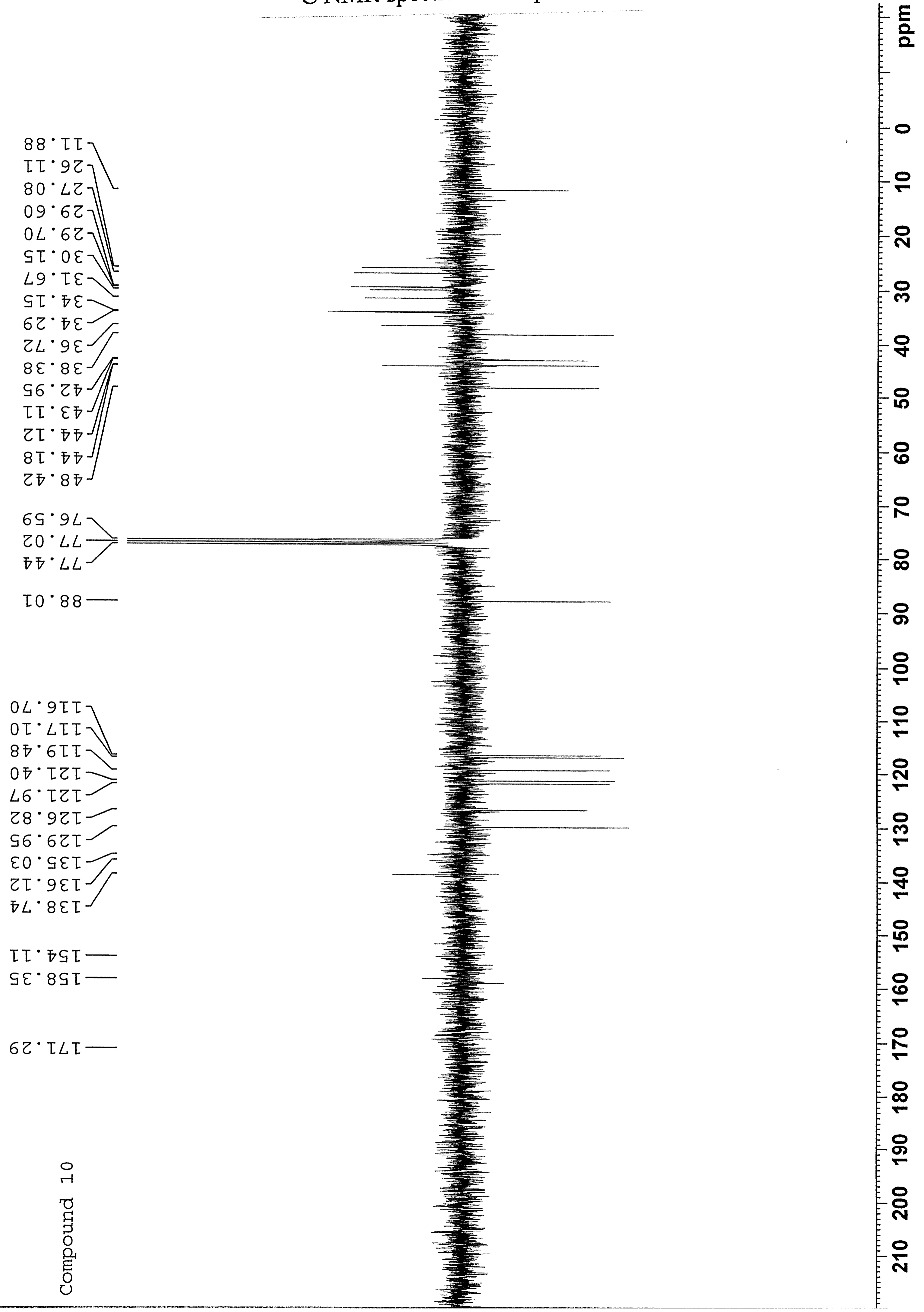

HRMS spectra of compound 10

Qualitative Compound Report

**Data File**	NGAD-593-178.d	**Sample Name**	NGAD-593-178
**Sample Type**	Sample	**Position**	Vial 11
**Instrument Name**	Instrument 1	**User Name**	Olivier Thibeault
**Acq Method**	Direct_Injection_ESI.m	**IRM Calibration Status**	[redacted]
**DA Method**	MFC.m	**Comment**	

Compound Table

Compound Label	RT	Mass	Abund	Formula	Tgt Mass	Diff (ppm)
Cpd 2: C28 H34 Br N O3	0.234	511.1712	11533	C28 H34 Br N O3	511.1722	-2.03

Compound Label	RT	Algorithm	Mass
Cpd 2: C28 H34 Br N O3	0.234	Find By Formula	511.1712

MS Spectrum

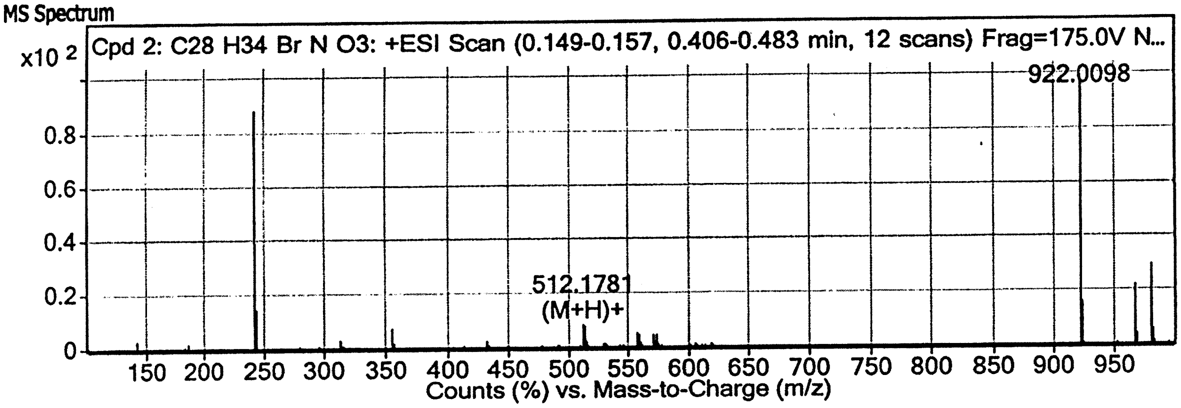

MS Zoomed Spectrum

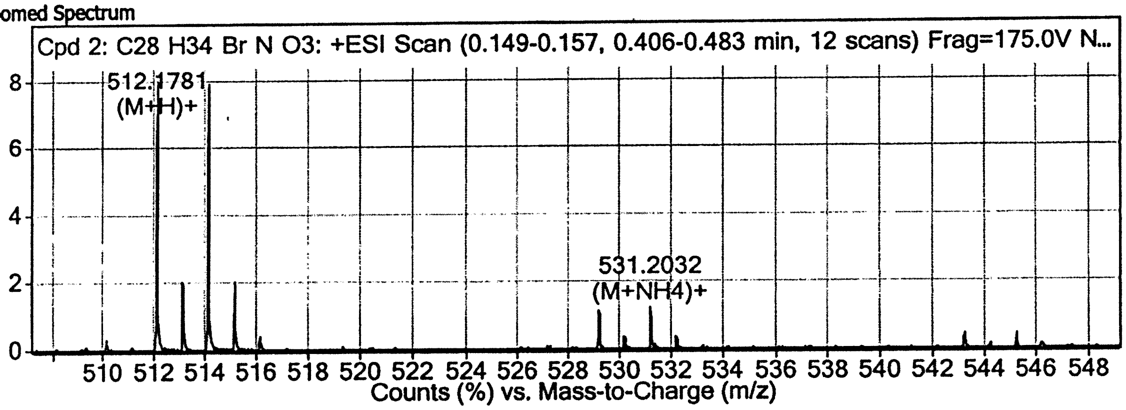

MS Spectrum Peak List

m/z	*Calc m/z*	Diff(ppm)	z	Abund	Formula	Ion
512.1781	512.1795	-2.74	1	11864	C28 H35 Br N O3	(M+H)+
513.182	513.1828	-1.59	1	3219	C28 H35 Br N O3	(M+H)+
514.1772	514.1779	-1.29	1	11626	C28 H35 Br N O3	(M+H)+
515.1802	515.1809	-1.4	1	3151	C28 H35 Br N O3	(M+H)+
529.2045	529.206	-2.85	1	1732	C28 H38 Br N2 O3	(M+NH4)+
531.2032	531.2044	-2.33	1	1792	C28 H38 Br N2 O3	(M+NH4)+
922.0098				146258		
922.392				5962		

HPLC chromatograms of compound **10**

==== Shimadzu LabSolutions Analysis Report ====

C:\LabSolutions\Data\Project1\EQUIPE DONALD POIRIER\Adrien NGAD\NGAD-593-179.lcd

Acquired by	: System Administrator
Sample Name	: NGAD-593-178
Sample ID	: NGAD-593-178
Tray#	: 1
Vial#	: 3
Injection Volume	: 8
Data File	: C:\LabSolutions\Data\Project1\EQUIPE DONALD POIRIER\Adrien NGAD\NG
Method File	: C:\LabSolutions\Data\Project1\EQUIPE DONALD POIRIER\Rene Maltais met
Report Format File	: C:\LabSolutions\Data\Project1\EQUIPE DONALD POIRIER\Rene Maltais (div
Month-Day Acquired	: 1/17/2022
Month-Day Processed	: 1/17/2022

<Chromatogram>

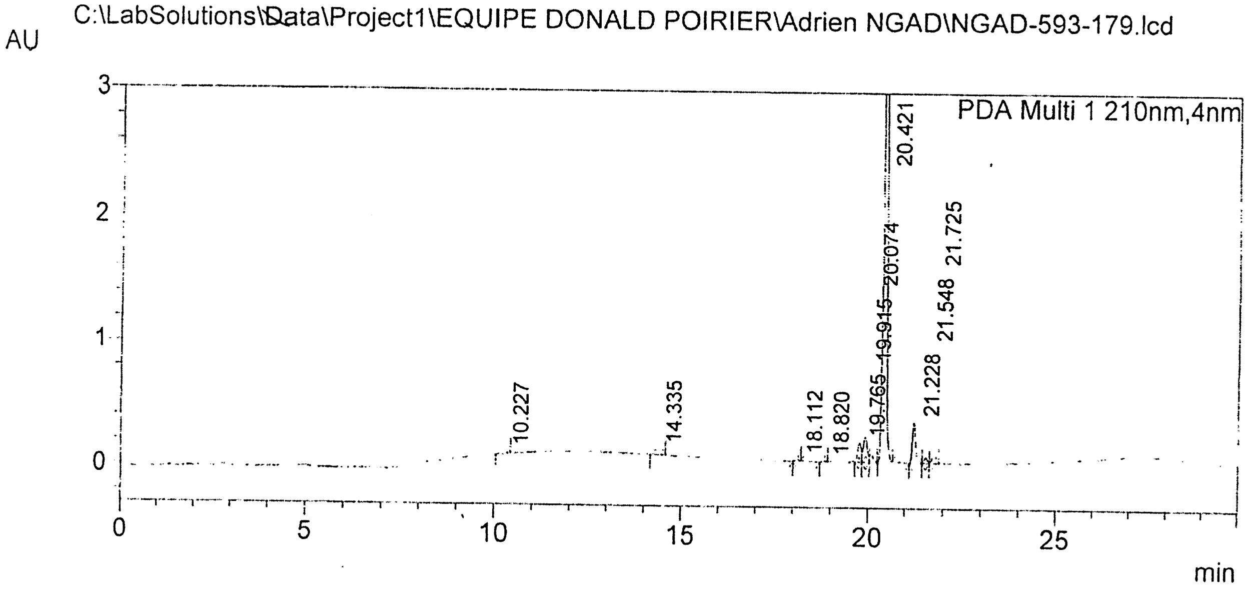

Peak Table

PDA Ch1 210nm

Peak#	Ret. Time	Area	Area%
1	10.227	237686	0.651
2	14.335	407080	1.114
3	18.112	86464	0.237
4	18.820	73672	0.202
5	19.765	958843	2.624
6	19.915	1440216	3.942
7	20.074	417695	1.143
8	20.421	30536866	83.574
9	21.228	1994418	5.458
10	21.548	227803	0.623
11	21.725	157776	0.432
Total		36538518	100.000
